# Magnetic Anisotropy of Homo- and Heteronuclear Terbium(III) and Dysprosium(III) Trisphthalocyaninates Derived from Paramagnetic ^1^H-NMR Investigation

**DOI:** 10.3390/molecules29020510

**Published:** 2024-01-19

**Authors:** Ilya D. Kormschikov, Marina A. Polovkova, Gayane A. Kirakosyan, Alexander G. Martynov, Yulia G. Gorbunova, Aslan Yu. Tsivadze

**Affiliations:** 1 Faculty of Chemistry, Lomonosov Moscow State University, GSP-1, Leninskie Gory, 119991 Moscow, Russia; kormshikovi@mail.ru; 2 Frumkin Institute of Physical Chemistry and Electrochemistry, Russian Academy of Sciences, Leninsky pr., 31, Building 4, 119071 Moscow, Russia; icemarin@mail.ru (M.A.P.); gayakira@mail.ru (G.A.K.); yulia@igic.ras.ru (Y.G.G.); tsiv@phyche.ac.ru (A.Y.T.); 3 Kurnakov Institute of General and Inorganic Chemistry, Russian Academy of Sciences, Leninsky pr., 31, 119991 Moscow, Russia

**Keywords:** phthalocyanine, triple-decker complex, heteronuclear complexes, terbium, dysprosium, solvation-induced switching, paramagnetic NMR, axial magnetic anisotropy

## Abstract

^1^H-NMR spectroscopy of lanthanide complexes is a powerful tool for deriving spectral–structural correlations, which provide a clear link between the symmetry of the coordination environment of paramagnetic metal centers and their magnetic properties. In this work, we have first synthesized a series of homo- (M = M* = Dy) and heteronuclear (M ≠ M* = Dy/Y and Dy/Tb) triple-decker complexes [(BuO)_8_Pc]M[(BuO)_8_Pc]M*[(15C5)_4_Pc], where BuO- and 15C5- are, respectively, butoxy and 15-crown-5 substituents on phthalocyanine (Pc) ligands. We provide an algorithmic approach to assigning the ^1^H-NMR spectra of these complexes and extracting the axial component of the magnetic susceptibility tensor, $\chi_{ax}$ . We show how this term is related to the nature of the lanthanide ion and the shape of its coordination polyhedron, providing an experimental basis for further theoretical interpretation of the revealed correlations.

## 1. Introduction

Single-molecule magnetism (SMM) is one of the most intriguing manifestations of the anisotropic coordination environment of paramagnetic lanthanides in sandwich complexes with tetrapyrrolic ligands, which was actually first described for this family of elements using the example of terbium(III) bisphthalocyaninate, **Tb(Pc)_2_**. Since the seminal report by Ishikawa et al. two decades ago [1], numerous examples of sandwiches containing mainly Tb^3+^ and Dy^3+^ metal centers have been synthesized and extensively studied in terms of magnetic relaxation dynamics, and the potential for fabrication of spintronic devices based on them has been clearly demonstrated [2,3,4].

Among such complexes, we can distinguish a prominent class of triple-decker phthalocyaninates, in which two metal ions bind three ligands, providing a wide range of combinations of different macrocycles and metal centers to achieve desired functional characteristics [5,6,7]. While the synthesis and properties of a large number of heteroleptic triple-deckers have been reported [8], the family of heteronuclear derivatives containing two different rare-earth elements (REEs) is still relatively underexplored. At the same time, such a combination provides unique possibilities for studying the subtle effects of intramolecular interactions between paramagnetic ions separated by a distance of only 3.4–3.5 Å.

The first vivid contribution to such studies was made by Ishikawa et al., who synthesized three series of heteroleptic triple-deckers [(BuO)_8_Pc]M*(Pc)M(Pc), where [(BuO)_8_Pc]^2−^ and (Pc)^2−^ were octa-butoxy-substituted and unsubstituted phthalocyaninato ligands, respectively [9,10]. One series consisted of homonuclear complexes with M* = M = Tb^3+^, Dy^3+^, Ho^3+^, Er^3+^, Tm^3+^ and Yb^3+^, and the other two families were isomeric heteronuclear complexes containing a diamagnetic Y^3+^ ion at either the M* or M site, while the other site was occupied by one of the listed lanthanides. A comparison of the temperature dependence of magnetic susceptibilities between homo- and heteronuclear complexes has provided the first detection and characterization of the dipolar *f*-*f* interactions between Ln^3+^ ions.

Later, the influence of these interactions on the behavior of SMMs based on triple-deckers was revealed. It was demonstrated that heteronuclear complexes containing one dia- and one paramagnetic ion, Tb^3+^ and Y^3+^, acted as field-induced SMMs, while the homonuclear di-terbium complexes showed slow magnetic relaxation even in a zero dc field. Such a behavior was attributed to the *f*-*f* interactions acting as an exchange bias that suppresses the undesired quantum tunneling mechanism of magnetic relaxation [11,12,13]. This effect was first demonstrated on the examples of the aforementioned [(BuO)_8_Pc]M*(Pc)M(Pc) complexes [14], and a deeper insight into this phenomenon was gained with the 15-crown-5-substituted sandwiches [(15C5)_4_Pc]M*[(15C5)_4_Pc]M(Pc) reported by R. Holmberg et al. [15]. In both cases, M* = M = Tb or M* ≠ M = Tb/Y.

**Figure 1:**
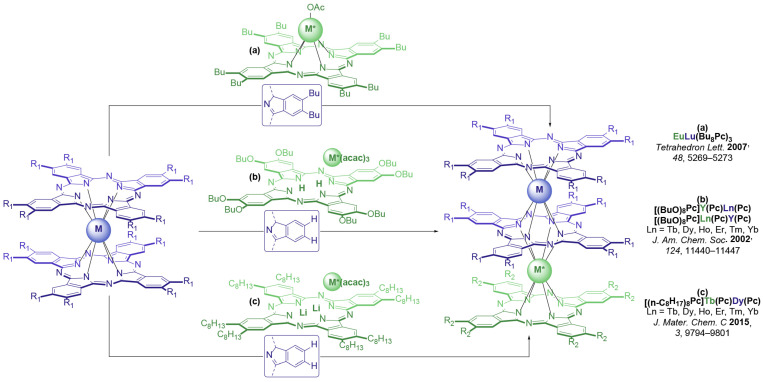
Summary of synthetic raise-by-one-story approaches to heteronuclear trisphthalocyaninates via addition of a pre-synthesized monophthalocyaninate to a double-decker complex (**a**) [16]; generation of monophthalocyaninates in situ from a metal-free ligand (**b**) [9]; and transmetalation of dilithium phthalocyaninate (**c**) [20].

The synthesis of heteronuclear trisphthalocyaninates is relatively straightforward. It implies raise-by-one-story reactions of bisphthalocyaninates bearing one metal center with monophthalocyaninates bearing another metal center. The latter precursors can be either synthesized beforehand [16,17,18] (Figure 1a), generated in situ from metal-free ligands [9] (Figure 1b) or by transmetalation of other complexes [15,19] (Figure 1c).

In general, these approaches can be used to obtain thermodynamically and kinetically stable heteronuclear triple-deckers with any pair of middle and late lanthanide ions, including complexes with two paramagnetic ions. Such an interplay between different combinations of nonequivalent magnetically active nuclei placed in well-defined positions can be used to study *f*-*f* interactions and provides a tool for fine-tuning the SMM behavior of such complexes. A rare example of such complexes—**[(*n*-C_8_H_13_)_8_Pc]Tb(Pc)Dy(Pc)**—has been reported by Lan et al. [20]. A weak but significant interaction between the two lanthanides was clearly detected, modifying the magnetic behavior of the single lanthanide as observed in the parent mononuclear complexes.

Trisphthalocyaninates also attract attention as conformationally flexible scaffolds where the twist angle between the ligands can be controlled by various inter- and intramolecular factors, including host–guest interactions [21,22], solvation [23,24] and redox transformations [25,26]. The variation in the twist angle inevitably changes the symmetry of the coordination polyhedron of the lanthanide ion sandwiched between these ligands, which has a profound effect on its magnetic properties due to the influence of this symmetry on magnetic anisotropy [27] and energetic characteristics and preferable mechanisms of magnetic relaxation [11,28,29].

**Figure 2:**
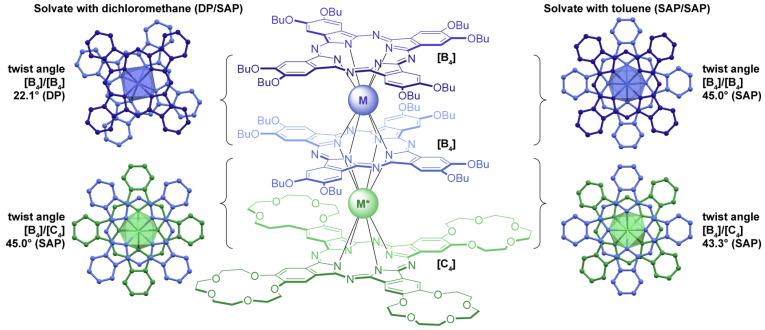
Conformationally flexible trisphthalocyaninates, [(BuO)_8_Pc]M[(BuO)_8_Pc]M*[(15C5)_4_Pc] (or [B_4_]M[B_4_]M*[C_4_] for brevity) capable of site-selective solvation-induced conformational switching. Pairs of neighboring ligands from X-ray structures of solvates of **[B_4_]Y[B_4_]Y[C_4_]** with dichloromethane (CCDC FIJTEB) and toluene (CCDC FIJXOP) show the square-antiprismatic and distorted prismatic conformers, respectively.

Recently, we reported a series of works on conformationally flexible triple-decker complexes composed of butoxy- and crown-substituted phthalocyaninato ligands [(BuO)_8_Pc]M[(BuO)_8_Pc]M*[(15C5)_4_Pc], including homonuclear complexes M = M* = Tb^3+^ and Y^3+^ [23] and their heteroleptic counterparts M ≠ M* = Tb^3+^ and Y^3+^ [24]. We showed that the pairwise conformations of the ligands are solvation-dependent: in halogenated alkanes, the pair of BuO- and 15C5-substituted ligands adopts the staggered conformation with a twist angle of ~45°, while the pair of BuO-substituted ligands forms the gauche conformation with a twist angle of ~22° (Figure 2). In turn, it provides metal centers M* and M with square-antiprismatic (SAP) and distorted prismatic (DP) coordination surroundings, respectively. In contrast, solvation with aromatic solvents leads to the stabilization of staggered conformations for both ligand pairs, so that both metal centers exist in SAP environments. The stabilization of certain conformers was explained by X-ray diffraction analysis of the solvates with dichloromethane and toluene, where weak contacts with the solvent molecules were found and analyzed in terms of the quantum theory of atoms in molecules [23]. Moreover, these conformations were stable not only in the crystalline state but also in solution, which was confirmed by ^1^H-NMR characterization of Tb^3+^ complexes—their magnetic anisotropy $\chi_{ax}$ strongly increases upon lowering the coordination symmetry from SAP to DP, as evidenced from the structure-based analysis of lanthanide-induced shifts.

In the present work, we extended the developed synthetic and analytical approaches to Dy^3+^ complexes, including a homonuclear triple-decker with M = M* = Dy^3+^ and pairs of isomeric heteronuclear analogues with M/M* = Dy^3+^/Y^3+^ and Y^3+^/Dy^3+^, as well as Dy^3+^/Tb^3+^ and Tb^3+^/Dy^3+^. For the sake of brevity, we will henceforth refer to butoxy- and crown-substituted ligands as **[B_4_]** and **[C_4_]**, respectively, following our previous notation [23,24] (Figure 2). Assuming that the axial anisotropy of the magnetic susceptibility tensor $\chi_{ax}$ deduced from NMR correlates with ligand field parameters [30,31,32,33,34], which, in turn, determine SMM behavior [26,35], these studies serve to verify the theoretical models and to design molecular magnets with improved characteristics [36].

## 2. Results

### 2.1. Synthesis and Characterization

**Figure 3:**
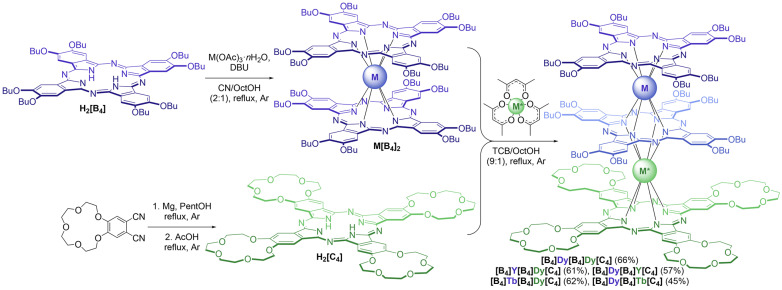
Synthesis of heteronuclear trisphthalocyaninates [B_4_]M[B_4_]M*[C_4_].

The synthesis of the [B_4_]M[B_4_]M*[C_4_] complexes with the aforementioned combinations of metal centers followed the procedure reported for Y^3+^ and Tb^3+^ complexes (Figure 3). It started with the preparation of bis(octa-*n*-butoxyphthalocyaninates) **M[B_4_]_2_**, M = Tb^3+^, Dy^3+^ and Y^3+^ by the interaction of **H_2_[B_4_]** with the corresponding metal acetates in a refluxing mixture of 1-chloronaphthalene (CN) and 1-octanol (OctOH) and DBU. Previously, we demonstrated that the use of this mixture is beneficial for the fast and efficient conversion of the starting ligand into metal complexes [23].

The resulting complexes underwent a reaction with tetra-15-crown-5-phthalocyanine, **H_2_[C_4_]**, in the presence of acetylacetonates M*(acac)_3_·*n*H_2_O in a refluxing mixture of 1,2,4-trichlorobenzene (TCB) and 1-octanol. Rapid conversion of the starting reagents into the desired triple-deckers was observed using UV-vis spectroscopy, and the target complexes were isolated by column chromatography on alumina. Due to the difference in polarity, [B_4_]M[B_4_]M*[C_4_] could be easily separated from the unreacted **M[B_4_]_2_** and the side homoleptic products M*_2_[C_4_]_3_.

**Figure 4:**
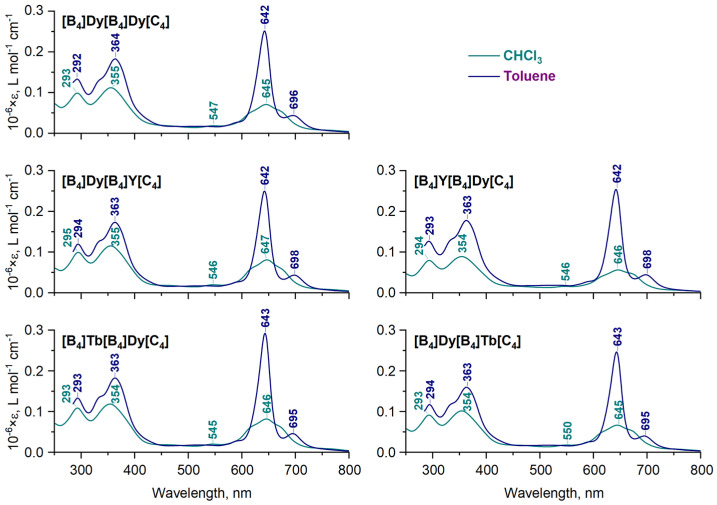
Comparison of the UV-vis spectra of homo- and heteronuclear complexes [B_4_]M[B_4_]M*[C_4_], M = M* = Dy, M ≠ M* = Dy/Y and M ≠ M* = Dy/Tb in chloroform and toluene.

UV-vis spectra measured in toluene and chloroform demonstrate that dysprosium-containing complexes exhibit pronounced solvatochromism, similarly to the previously reported Tb- and Y-containing [B_4_]M[B_4_]M*[C_4_] complexes [23,24] (Figure 4). In particular, the spectra of the newly synthesized complexes in toluene contain intense Q-bands with well-resolved splitting (642–643 and 695–698 nm) and less intense Soret and N-bands at 363–364 and 292–294 nm, while in the spectra in chloroform, the Q-bands are severely broadened, although the maxima of the Q-bands in the two different solvents are very close. This has been previously explained by the difference in the conformational state of molecules, as in the spectrum of the conformer with DP/SAP coordination polyhedral, more electronic transitions are symmetrically allowed in comparison with the SAP/SAP conformer [25,37]. Since the positions and splitting of Q-bands in the UV-vis spectra of sandwich complexes are governed by the interligand distances, which in turn depend on the size of the metal centers [19,37,38], the faintly small difference in the spectral appearances of the synthesized complexes in each of the solvents is explained by the similarity of the radii of Y^3+^, Tb^3+^ and Dy^3+^ ions [39].

The very weak absorption of lanthanide ions in the synthesized complexes could not be detected due to the much stronger absorption of tetrapyrrolic ligands. Also, we could not detect *f*-luminescence of Tb^3+^ and Dy^3+^ ions due to reabsorption of weak lanthanide-centered emission by tetrapyrrolic ligands, which have absorption bands with extinction coefficients of 1.4−2.0 × 10^4^ L mol^−1^ cm^−1^ in the range of 400–600 nm, where the emission of these ions is typically observed in coordination compounds with colorless or weakly absorbing ligands [40]. Nevertheless, there are reports of *f*-luminescence observed in the near-IR for Nd(III), Ho(III), Er(III) and Yb(III) and complexes where Pcs and related macrocycles typically do not absorb light [41].

While optical methods are not suitable to confirm the presence of lanthanides in complexes with phthalocyanine ligands, the chemical composition of the synthesized triple*-*deckers is unambiguously confirmed by MALDI-TOF mass spectrometry due to the good agreement between the calculated and experimentally observed isotopic distributions (Figures S1–S5). The exact arrangement of the ligands and metal centers is determined by NMR spectroscopy, as discussed in the following sections.

### 2.2. Analysis of Lanthanide-Induced Shifts in ^1^H-NMR Spectra

The ^1^H-NMR spectra of lanthanide complexes typically have strikingly different appearances from the spectra of diamagnetic organic compounds since the presence of paramagnetic metal centers causes up- or downfield shifts of resonance signals by tens or even hundreds of ppm. The signs and magnitudes of these lanthanide-induced shifts (LISs) depend on both the nature of the lanthanide ion [42,43,44,45] and the overall geometry of the complex [46,47].

Thus, for the *k*-th proton, the LIS can be expressed as the difference between its chemical shifts in isostructural para- and diamagnetic complexes (1):

(1) $$\Delta \delta_{k}=\delta_{k}^{para}-\delta_{k}^{dia}$$

On the other hand, the LIS can be presented as the sum of typically negligible contact (through-bond, $\delta_{k}^{con}$ ) and predominant dipolar (through-space, $\delta_{k}^{dip}$ ) contributions:

(2) $$\Delta \delta_{k}=\delta_{k}^{con}+\delta_{k}^{dip}\approx \frac{\chi_{ax}}{12\pi }\cdot G_{k,}\;\;G_{k}=\frac{3\cos^{2}\theta_{k}-1}{r_{k}^{3}}$$

**Figure 5:**
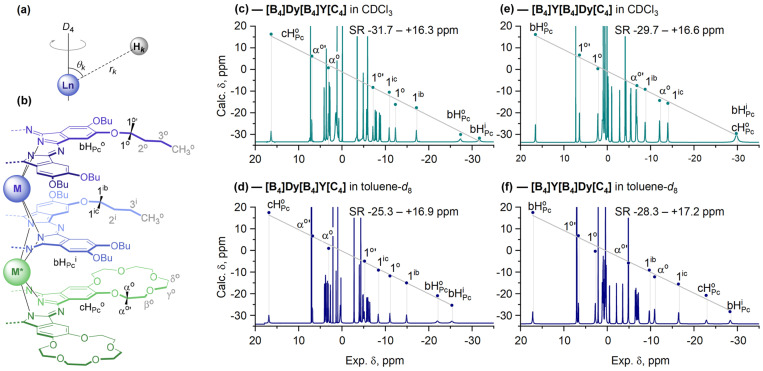
Explanation of values of $r_{k}$ and $\theta_{k}$ included in the geometric parameter $\;G_{k}$ according to Equation (2) (**a**). Labels of protons: black labels indicate protons used for further analysis (**b**). ^1^H-NMR spectra of **[B_4_]Dy[B_4_]Y[C_4_]** (**c**,**d**) and **[B_4_]Y[B_4_]Dy[C_4_]** (**e**,**f**) in CDCl_3_ (upper row) and toluene-*d*_8_ (lower row). Dots show the positions of chemical shifts calculated with Equation (4) vs. the experimental values (axes *x*). Grey lines show the least-squares fits between the calculated and experimental values. The complete assignment of NMR spectra for all complexes is presented in the Materials and Methods section and the Electronic Supporting Information (Figures S7–S14).

Here, $G_{k}$ is a geometrical parameter depending on the distance $r_{k}$ between the *k*-th proton and the lanthanide ion, and $\theta_{k}$ is the angle between the vector ( $\bar{H_{k};{\mathrm{Ln}}^{3+}}$ ) and the main symmetry axis, D_4_ in our case (Figure 5a). The spectral–structural correlation (2) renders lanthanide ions as perfect probes for the elucidation of the solution structures, for example, in structural biology [48,49].

The proportionality factor $\chi_{ax}$ is the axial component of the magnetic susceptibility tensor. Thus, the observation of lanthanide-induced shifts in NMR spectra is a manifestation of lanthanide magnetic properties associated with crystal field parameters [42,43,44,46,47], and the value of $\chi_{ax}$ complements the easily affordable NMR with much more sophisticated time- and resource-consuming magnetochemical measurements [10,31,50,51,52,53].

The dipolar approximation of the LIS using Equation (2) suggests that the ratio of LISs for the pair of *k*-th and *l*-th protons can be approximated with the ratio of their geometrical parameters, $R_{kl}$ :

(3) $$\frac{\Delta \delta_{k}}{\Delta \delta_{l}}\approx R_{kl},\;R_{kl}=\frac{G_{k}}{G_{l}}$$

A combination of Equations (1) and (3) written for a pair of protons gives Equation (4), which can be used to estimate the positions of the resonance signals in the ^1^H-NMR spectra of paramagnetic complexes using the spectrum of a diamagnetic complex with a well-established structure and the shift of at least one firmly assigned resonance signal in the spectrum of a paramagnetic complex:

(4) $$\delta_{k}^{para}\approx \delta_{k}^{dia}+\Delta \delta_{l}\cdot R_{kl}$$

Yttrium(III) complexes are typically used as diamagnetic references, providing $\delta_{k}^{dia}$ values, and the ratio of geometrical parameters $R_{kl}$ can be taken from the structural models obtained from either X-ray characterization or DFT modeling [22].

The almost perfect agreement of the UV-vis spectra of the newly synthesized complexes with those reported previously (Figure 4) suggests an analogy in the solution structure of the entire series of [B_4_]M[B_4_]M*[C_4_], which is generally expected given the close values of the ionic radii of Y^3+^ (1.019 pm), Tb^3+^ (1.040 pm) and Dy^3+^ (1.027 pm) [39], together with the similarity of their coordination chemistry [4]. This conclusion justifies the following application of the previously determined X-ray structures of **[B_4_]Y[B_4_]Y[C_4_]** solvated either with dichloromethane (DP/SAP conformer) or with toluene (SAP/SAP conformer) [23] for the analysis of ^1^H-NMR spectra of paramagnetic complexes containing Tb^3+^ and Dy^3+^ ions using Equation (4).

It is worth noting that the X-ray structures of the solvates with CHCl_3_ are not yet available, although we attempted to grow single crystals by diffusion of acetonitrile, heptane or vapors of pentane into **[B_4_]Y[B_4_]Y[C_4_]** solutions in CHCl_3_ but failed to obtain material of sufficient quality to perform XRD experiments with the required precision. On the other hand, we previously demonstrated that chloroform also stabilizes staggered and gauche conformers of 15C5- and BuO-substituted triple-deckers, similarly to dichloromethane (see Figure S6 for more details). Moreover, we previously showed that the spectra of **[B_4_]Tb[B_4_]Tb[C_4_]** in CDCl_3_ and CD_2_Cl_2_ are almost indistinguishable [23]. Altogether, this suggests that the X-ray structure of the DP/SAP conformer solvated with CH_2_Cl_2_ is a reasonable structural model for the analysis of LISs measured in chloroform.

#### 2.2.1. Analysis of Lanthanide-Induced Shifts in ^1^H-NMR Spectra of Heteronuclear Complexes **[B_4_]Dy[B_4_]Y[C_4_]** and **[B_4_]Y[B_4_]Dy[C_4_]**

The ^1^H-NMR spectra of the pair of isomeric trisphthalocyaninates **[B_4_]Dy[B_4_]Y[C_4_]** and **[B_4_]Y[B_4_]Dy[C_4_]** measured in CDCl_3_ and toluene-*d*_8_ evidence that the spectral range (SR) from the most upfield-shifted to the most downfield-shifted signals depends on both the position of the Dy^3+^ ion and the solvent applied (Figure 5b,c).

Only aromatic protons and methylene protons proximal to the Pc ligands—1^i,o^-CH_2_ groups of butoxy-substituents and α^o^-CH_2_ groups of crown-ether rings (black labels in Figure 5b)—were taken for further analysis as they refer to the most rigid part of the molecules, assuring the consistency between the X-ray and solution structures [23,24]. Although CH_2_ groups can rotate around single σ-bonds, the interconversion between the *exo*- and *endo*-H protons of the considered methylenes is slow on the NMR timescale; their resonances appear as pairs of signals separated by ca. 10 ppm and coupled in ^1^H-^1^H COSY spectra. Such hindered rotation can be explained by numerous weak interactions of the aliphatic substituents with the substituents in the neighboring ligands [23] and solvent molecules [25]. Similar splitting patterns were reported previously by Enders and Yamashita et al. [26,54], Jiang et al. [55] and Ishikawa et al. [56], together with our publications [19,23,24].

The splitting is much smaller for more distant methylene groups (grey labels in Figure 5b), and it almost vanishes for the 3^i,o^-CH_2_ and CH_3_ groups, suggesting that these groups rotate nearly freely around the corresponding sigma bonds. Thus, these distant protons were not taken into consideration so that the deduced $\chi_{ax}$ value is not disturbed by the conformational flexibility of the peripheral substituents. Good correlations between the experimentally observed chemical shifts and the shifts calculated from X-ray data (Figure 5c–f) validate the applied assumptions and approximations.

**Figure 6:**
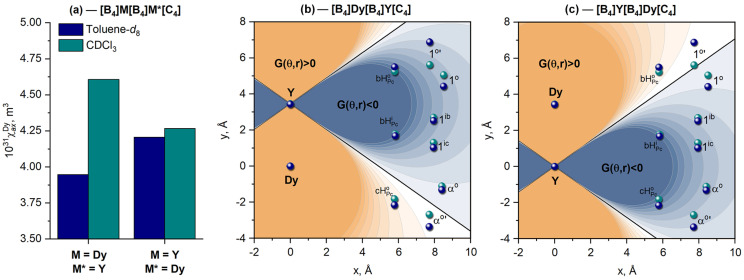
Values of axial anisotropy $\chi_{ax}$ found by least-squares linearization of $\Delta \delta_{k}$ vs. $G_{k}$ according to Equation (2) for Dy^3+^ ions in different surroundings (**a**). Contour maps of the $G\left(\theta ,r\right)$ function plotted for **[B_4_]Dy[B_4_]Y[C_4_]** (**b**) and **[B_4_]Y[B_4_]Dy[C_4_]** (**c**); the coordinates of selected protons were taken from the X-ray structures of the corresponding solvates with dichloromethane (cyan balls) and toluene (dark-blue balls). Labels of protons are given in Figure 5b.

The multidirectional nature of LISs observed in Figure 5c–f in such complexes originates from the fact that different protons have either positive or negative $G_{k}$ depending on the location of these protons in the principle magnetic framework of the Dy^3+^ ion (Figure 6b,c), and the largest absolute value of $G_{k}$ deduced from X-ray data corresponds to the aromatic proton of the inner BuO-substituted ligand bH_Pc_^i^ [24]. This observation allowed us to assign the resonance signals of the other protons using Equation (4), which was confirmed by further ^1^H-^1^H COSY spectra (Figures S7–S14).

Plotting LIS from NMR spectra vs. the geometrical parameters found from X-ray data, followed by linear regression analysis, afforded axial anisotropies of Dy^3+^ ions in different coordination surroundings (Figure 6a). This analysis demonstrates that the magnetic properties of this ion are sensitive to the symmetry of the coordination surrounding. Thus, switching from an SAP to a DP polyhedron in toluene and chloroform, respectively, causes a notable increase in axial anisotropy, similar to the results reported previously for Tb^3+^ ions in isostructural complexes.

#### 2.2.2. Analysis of Lanthanide-Induced Shifts in ^1^H-NMR Spectra of the Homonuclear Complex **[B_4_]Dy[B_4_]Dy[C_4_]**

Due to the presence of two structurally nonequivalent paramagnetic centers in **[B_4_]Dy[B_4_]Dy[C_4_]**, Equation (4) cannot be applied for the assignment of its ^1^H-NMR spectra, as these centers might give different contributions to the total LIS value. Therefore, in the present case, the assignment is made by approximating the chemical shift of the *k*-th proton in the homonuclear complex with the sum of the shifts of analogous protons in the heteronuclear complexes according to Equation (5) [56].

(5) $$\delta_{k}^{\left[B_{4}]Dy[B_{4}]Dy[C_{4}\right]}\approx \delta_{k}^{\left[B_{4}]Dy[B_{4}]Y[C_{4}\right]}+\delta_{k}^{\left[B_{4}]Y[B_{4}]Dy[C_{4}\right]}$$

The assignment is confirmed by ^1^H-^1^H COSY. In addition, due to the proximity of all the considered protons to the Dy^3+^ ions, their signals are the most broadened, providing additional verification of their correct assignment.

**Figure 7:**
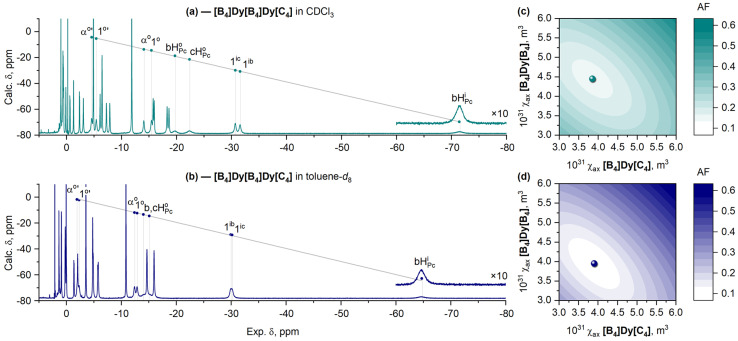
^1^H-NMR spectra of **[B_4_]Dy[B_4_]Dy[C_4_]** in CDCl_3_ (**a**) and toluene-*d*_8_ (**b**). Dots show the chemical shifts calculated with Equation (5) (axes *y*) vs. the experimental values (axes *x*). Grey lines show the least-squares fits between the calculated and experimental values. Labels of protons are given in Figure 5c. Graphical search for values of $\chi_{ax}$ for Dy^3+^ cations at **[B_4_]**/**[C_4_]** (axes *x*) and **[B_4_]**/**[B_4_]** (axes *y*) sites corresponding to minimal values of the agreement factor, AF, in CDCl_3_ (**c**) and toluene-*d*_8_ (**d**). Labels of protons are given in Figure 5b. The complete assignment of NMR spectra for all complexes is presented in the Materials and Methods section and the Electronic Supporting Information (Figures S15–S18).

A comparison of the ^1^H-NMR spectra of the homonuclear complex **[B_4_]Dy[B_4_]Dy[C_4_]** measured in CDCl_3_ and toluene-*d*_8_ (Figure 7a,b) suggests that switching from the SAP/SAP conformer to the DP/SAP conformer also causes an increase in anisotropy, as evidenced by the broadening of the spectral range. To confirm this conclusion, we assumed that each of the Dy^3+^ ions has its own $\chi_{ax}$ value in accordance with Equation (6); thus, we used two-dimensional minimization of the Wilcott factor (AF, Equation (7)) across the various pairs of $\chi_{ax}$ to find the best agreement between the calculated and experimental LIS values (Figure 7c,d).

(6) $$\Delta \delta_{k}^{\left[B_{4}]Dy[B_{4}]Dy[C_{4}\right]}\approx {\left[\frac{\chi_{ax}}{12\pi }\cdot G_{k}\right]}^{\left[B_{4}]Dy[B_{4}]Y[C_{4}\right]}+{\left[\frac{\chi_{ax}}{12\pi }\cdot G_{k}\right]}^{\left[B_{4}]Y[B_{4}]Dy[C_{4}\right]}$$

(7) $$AF=\sqrt{\frac{\sum_{k}{\left(\delta_{k}^{calc}-\delta_{k}^{exp}\right)}^{2}}{\sum_{k}{\delta_{k}^{exp}}^{2}}}$$

**Figure 8:**
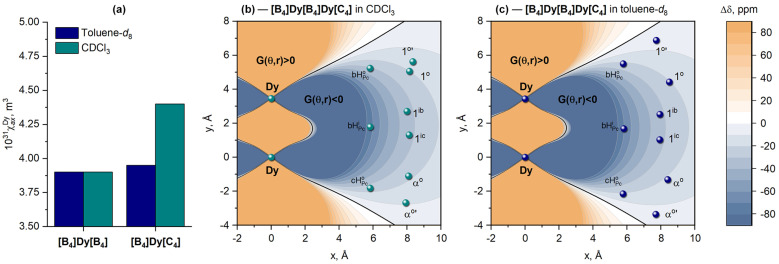
Values of axial anisotropy $\chi_{ax}$ for the Dy^3+^ ions at two different sites of **[B_4_]Dy[B_4_]Dy[C_4_]** in toluene-*d*_8_ and CDCl_3_ (**a**). Contour maps of the net LIS functions according to Equation (6) plotted for **[B_4_]Dy[B_4_]Dy[C_4_]** in CDCl_3_ (**b**) and toluene-*d*_8_ (**c**). Labels of protons are given in Figure 5b.

The results presented in Figure 7 suggest that the change in anisotropy in the homonuclear complex **[B_4_]Dy[B_4_]Dy[C_4_]** follows the same trends observed in heteronuclear complexes. In particular, switching between SAP/SAP (in toluene-*d*_8_) and DP/SAP (in CDCl_3_) causes an increase in the anisotropy of the ion in the conformationally flexible **[B_4_]/[B_4_]** site from 3.95 × 10^−31^ to 4.28 × 10^−31^ m^3^, while the $\chi_{ax}$ value of the Dy^3+^ ion in the conformationally invariant **[B_4_]**/**[C_4_]** site remains almost the same—3.9 × 10^−31^ m^3^ (Figure 8a).

Plotting the contour maps of $\Delta \delta_{k}^{para}$ for the molecules of the **[B_4_]Dy[B_4_]Dy[C_4_]** complex in CDCl_3_ and toluene-*d*_8_ using the derived $\chi_{ax}$ values explains why all signals in the spectra have shifts of the same sign—all protons fall into the region with the same sign of the net $G\left(\theta ,r\right)$ function, in contrast to the heteronuclear derivatives, where protons fall into regions with different signs of the geometrical parameter (Figure 8b,c).

#### 2.2.3. Analysis of Lanthanide-Induced Shifts in ^1^H-NMR Spectra of Heteronuclear Complexes **[B_4_]Dy[B_4_]Tb[C_4_]** and **[B_4_]Tb[B_4_]Dy[C_4_]**

**Figure 9:**
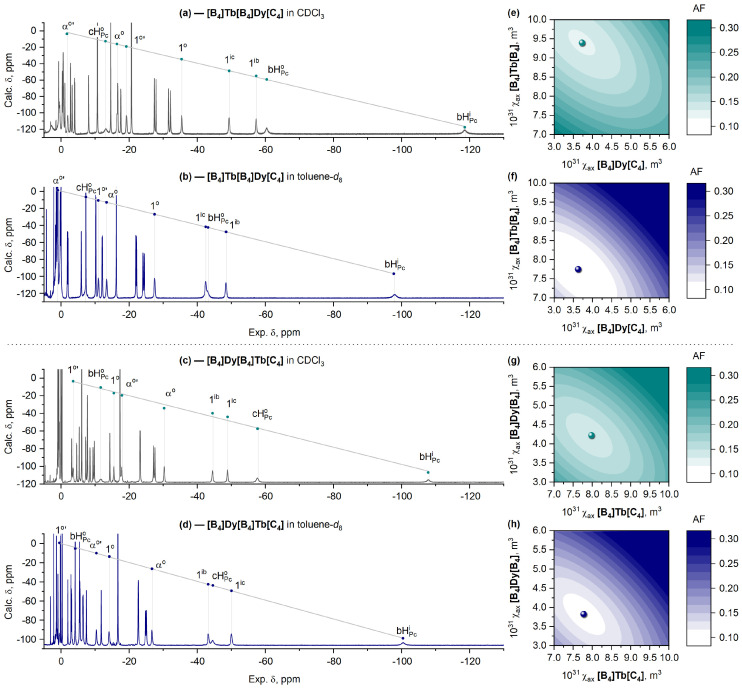
^1^H-NMR spectra of **[B_4_]Tb[B_4_]Dy[C_4_]** and **[B_4_]Dy[B_4_]Tb[C_4_]** in CDCl_3_ (**a**,**c**) and toluene-*d*_8_ (**b**,**d**). Dots show the chemical shifts calculated with Equation (5) (axes *y*) vs. the experimental values (axes *x*). Grey lines show the least-squares fits between the calculated and experimental values. Labels of protons are given in Figure 5b. Graphical search for values of $\chi_{ax}$ for corresponding lanthanide cations at the **[B_4_]**/**[C_4_]** (axes *x*) and **[B_4_]**/**[B_4_]** (axes *y*) sites corresponding to minimal values of the agreement factor, AF, in CDCl_3_ (**e**,**g**) and toluene-*d*_8_ (**f**,**h**). The complete assignment of NMR spectra for all complexes is presented in the Materials and Methods section and the Electronic Supporting Information (Figures S19–S26).

Following the procedure described above, we assigned and analyzed the spectra of two isomeric complexes, **[B_4_]Dy[B_4_]Tb[C_4_]** and **[B_4_]Tb[B_4_]Dy[C_4_]**, in CDCl_3_ and toluene-*d*_8_ (Figure 9) to trace the change in the axial anisotropy of both lanthanide ions simultaneously using Equations (5) and (6). The assignment was confirmed by ^1^H-^1^H COSY (Figures S13–S16).

**Figure 10:**
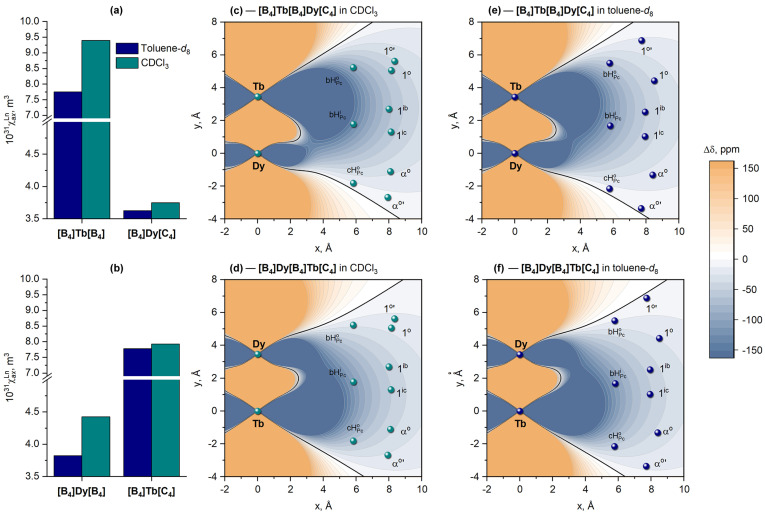
Values of axial anisotropy $\chi_{ax}$ for Tb^3+^ and Dy^3+^ ions in heteronuclear complexes **[B_4_]Tb[B_4_]Dy[C_4_]** (**a**) and **[B_4_]Dy[B_4_]Tb[C_4_]** (**b**). Contour maps of the net LIS functions according to Equation (6) plotted for **[B_4_]Tb[B_4_]Dy[C_4_]** (**c**,**d**) and **[B_4_]Dy[B_4_]Tb[C_4_]** (**e**,**f**) in CDCl_3_ (**a**,**c**) and toluene-*d*_8_ (**b**,**d**). Labels of protons are given in Figure 5b.

It can be clearly seen that the change from CDCl_3_ to toluene-*d*_8_ has the most pronounced influence on the ^1^H-NMR spectra of **[B_4_]Tb[B_4_]Dy[C_4_]**, where the Tb^3+^ ion is placed into the switchable **[B_4_]/[B_4_]** site (Figure 9a,b), which is expectedly followed by a significant decrease in its axial anisotropy—from 9.40 × 10^−31^ to 7.75 × 10^−31^ m^3^—in agreement with the behavior of the previously studied Tb^3+^ complexes [23,24]. Switching the coordination polyhedron of the Dy^3+^ ion from DP to SAP also causes a decrease in axial anisotropy (Figure 9c,d), although this effect is not so strong compared to the Tb^3+^ metal centers (Figure 10a,b).

Finally, the availability of $\chi_{ax}$ for each metal center allows plotting the contour maps of the overall LIS values for different conformers of **[B_4_]Tb[B_4_]Dy[C_4_]** and **[B_4_]Dy[B_4_]Tb[C_4_]** (Figure 10c–f). These plots vividly explain the difference between the spectral appearances of these complexes and their homonuclear analogues. For example, it can be seen that a combination of two metal ions with essentially different anisotropies results in deformation of the zero Δδ isosurface, which approaches the protons of the terminal ligands bound to the Dy^3+^ ion; this is clearly seen in the experimental spectra, in which the less downfield-shifted signals refer to aromatic protons (either bH_Pc_^o^ or cH_Pc_^o^) and *exo*-protons of methylene groups (1^o^′ or α^o^′, respectively).

## 3. Discussion

The presented results give several guidelines for further work on lanthanide complexes with phthalocyanine ligands:

- The trisphthalocyanine scaffold affords the synthesis of heteronuclear complexes with a precise arrangement of rare-earth ions due to its thermodynamical and kinetic stability. Complexes with different combinations of paramagnetic lanthanides can be efficiently obtained, which provides the basis for further investigation of intramolecular *f*-*f* interactions and the elaboration of molecular magnetic materials. In the present work, the combinations of two strongly paramagnetic Tb^3+^ and Dy^3+^ ions were used to obtain isomeric heteronuclear complexes, but, obviously, other combinations of middle and late lanthanides can also be used in this type of chemistry to obtain complexes with the required number of unpaired *f*-electrons.
- The comprehensive ^1^H-NMR spectroscopic characterization of strongly paramagnetic complexes is based on the appropriate structural model; therefore, this work provides algorithms for dealing with the spectra of complexes containing either one or two lanthanide ions, which are not necessarily equivalent. In this regard, the results presented demonstrate that the application of paramagnetic ^1^H-NMR spectroscopy should not be limited to routine identification but can be used to extract the magnetic properties of lanthanide ions [53]. In this context, our report follows the strategies applied by Enders and Yamashita, where Tb(III) and Dy(III) sandwich phthalocyaninates were comprehensively studied using NMR spectroscopy [26,35,54,57,58], and the influence of electronic and structural effects on their magnetic properties, especially $\chi_{ax}$ , was revealed. In summary, it is expected that further magnetochemical studies of the newly synthesized lanthanide phthalocyaninates will provide more correlations between the $\chi_{ax}$ term and the energetic properties of slow magnetic relaxation.
- The addition of controllable conformational flexibility gives one more degree of freedom to control the magnetic properties of sandwich lanthanide complexes. The previously discovered correlations between the symmetry of the coordination polyhedron of the Tb^3+^ ion and its magnetic properties are also valid for the Dy^3+^ ion, and the effect of the conformational switching can be studied for other lanthanides to find the capabilities and limitations of theoretical models.
- Importantly, our study evidences that the $\chi_{ax}$ values for Dy^3+^ are nearly twice smaller than those for Tb^3+^; however, this observation does not match the expectations from Bleaney’s theory, where the largest anisotropy in the lanthanide series is expected for dysprosium [43]. Moreover, it contradicts the results of theoretical modeling obtained by Mironov et al. [32] for various polyhedra of lanthanide complexes, where the most pronounced influence of the surrounding coordination was anticipated for dysprosium complexes. The reason for this discrepancy may be a violation of the theory’s basic assumption that the thermal energy is larger than the ligand field splitting; thus, further theoretical modeling using ab initio methods might be particularly helpful [59,60].

## 4. Materials and Methods

### 4.1. Materials

Starting phthalocyanines **H_2_[B_4_]**, **Y[B_4_]_2_**, **Tb[B_4_]_2_**, **Dy[B_4_]_2_** and **H_2_[C_4_]** were synthesized according to the previously reported procedures [61,62]. 1,2,4-Trichlorobenzene (TClB, for synthesis, 1-octanol (for synthesis), rare-earth acetylacetonates (Sigma-Aldrich, Burlington, MA, USA), and neutral alumina (50–200 μm, Macherey-Nagel, Düren, Germany) were used as received from the commercial suppliers. Chloroform (reagent grade, Ekos-1, Staraya Kupavna, Russia) was distilled over CaH_2_.

### 4.2. Methods

Matrix-assisted laser desorption ionization time-of-flight (MALDI-TOF) mass spectra were measured on a Bruker Daltonics Ultraflex spectrometer. Mass spectra were registered in positive ion mode using 2,5-dihydroxybenzoic acid as a matrix. UV-vis spectra in the range of 250–900 nm were measured using a Thermo Evolution 210 spectrophotometer in quartz cells with 0.5–1.0 cm optical path lengths.

^1^H NMR spectra were recorded at 303 K on a Bruker Avance III 600 MHz spectrometer equipped with a 5 mm Z-gradient BBO probe (*zg30* pulse program from Topspin library). A total of 128 scans with a 30° excitation pulse (90° pulse width was 13.8 μs, DE 6.50 μs) and 1 s delay were accumulated for each system. Spectral width was taken to be 90–100 kHz, depending on the combination of lanthanides. Gaussian multiplication was used for processing. Automatic polynomial baseline correction was applied. For recording 2D COSY spectra (*cosygpqf* program from Topspin library), spectral width was selected to be 40–75 ppm, depending on the combination of lanthanides. The residual solvent resonances were used as internal references (δ toluene 7.09 ppm, chloroform 7.26 ppm). Typically, 5 mg of complexes was dissolved in 0.6 mL of a corresponding deuterated solvent to provide a concentration of ca. 2.3 mM. The applied deuterated chloroform (99.8 atom% D, ZEOchem, Uetikon am See, Switzerland) was filtered prior to use through Pasteur pipettes filled with alumina to remove possible acidic impurities. Deuterated toluene (99.8 atom% D, ABCR, Karlsruhe, Germany) was used without additional purification.

### 4.3. Synthesis and Characterization of the Triple-Decker Complexes

***Trisphthalocyaninate [B_4_]Dy[B_4_]Y[C_4_]:*** A solution of phthalocyanines **Dy[B_4_]_2_** (30.0 mg, 12.8 μmol) and **H_2_[C_4_]** (20.4 mg, 16.0 μmol) in a mixture of 2.7 mL 1,2,4-trichlorobenzene and 0.3 mL n-octanol was heated to reflux at 230 °C under a stream of argon, and solid yttrium (III) acetylacetonate hydrate Y(acac)_3_·*n*H_2_O (19.4 mg, 48.1 μmol) was added. After 10 min, the reaction mixture was cooled to ambient temperature. The reaction mixture was transferred to a chromatographic column packed with alumina in a mixture of CHCl_3_ and hexane (1:1 *v*/*v*). The target complex was isolated by elution with a CHCl_3_-hexane mixture (4:1 *v*/*v*), followed by a mixture of CHCl_3_ with 0→1% MeOH as a dark-blue solid (29 mg, yield 61%).

MALDI TOF: *m*/*z* calculated for C_192_H_232_DyN_24_O_36_Y 3703.5, found 3704.2 [M^+^].

UV-vis (CHCl_3_) λ_max_ (nm) (log ε): 647 (5.06), 546 (4.50), 355 (5.21), 295 (5.16).

UV-vis (Toluene) λ_max_ (nm) (log ε): 698 (4.64), 642 (5.40), 363 (5.24), 294 (5.09).

^1^H-NMR (600 MHz, CDCl_3_) δ −31.71 (br s, 8H, bH_Pc_^i^), −27.34 (br s, 8H, bH_Pc_^o^), −17.16 (br s, 8H, 1^ib^), −12.32 (br s, 8H, 1^o^), −10.87 (br s, 8H, 1^ic^), −8.88 (s, 8H, 2^ib^), −8.62 (s, 8H, 2^ic^), −7.92 (s, 8H, 3^ib^), −7.65 (s, 8H, 3^ic^), −7.14 (br s, 8H, 1^o^′), −5.91 (s, 32H, CH_3_^i^ + 2^o^), −5.69 (s, 8H, 2^o^′), −4.89 (s, 16H, 3^o,o^′), −3.50 (s, 24H, CH_3_^o^), 2.77 (s, 8H, β^o^), 2.83 (m, 8H, γ^o^), 2.96 (m, 8H, γ^o^′), 3.13 (d, 25 Hz, 8H, α^o^), 3.62 (m, 16H, δ^o,o^′), 4.13 (s, 8H, β^o^′), 6.95 (d, 25 Hz, 8H, α^o^′), 16.34 (br s, 8H, cH_Pc_^o^).

^1^H-NMR (600 MHz, Toluene-*d*_8_) δ −25.33 (br s, 8H, bH_Pc_^i^), −22.01 (br s, 8H, bH_Pc_^o^), −14.89 (br s, 8H, 1^ib^), −11.06 (br s, 8H, 1^o^), −8.37 (br s, 8H, 1^ic^), −6.40 (s, 8H, 2^ib^), −6.07 (s, 8H, 2^ic^), −5.95 (s, 8H, 3^ib^), −5.70 (s, 8H, 3^ic^), −5.12 (s, 8H, 1^o^′), −4.88 (s, 8H, 2^o^), −4.80 (s, 8H, 2^o^′), −4.33 (br t, 24H, CH_3_^i^), −3.99 (s, 16H, 3^o,o^′), −2.79 (br t, 24H, CH_3_^o^), 2.68 (s, 8H, β^o^), 3.08 (d, 30 Hz, 8H, α^o^), 3.18 (br m, 8H, γ^o^), 3.62 (br m, 8H, δ^o^), 3.74 (br m, 8H, γ^o^′), 3.81 (s, 8H, β^o^′), 4.03 (s, 8H, δ^o^′), 6.81 (d, 30 Hz, 8H, α^o^′), 16.86 (br s, 8H, cH_Pc_^o^).

***Trisphthalocyaninate [B_4_]Y[B_4_]Dy[C_4_]:*** A solution of phthalocyanines **Y[B_4_]_2_** (23.0 mg, 10.0 μmol) and **H_2_[C_4_]** (16.0 mg, 12.6 μmol) in a mixture of 2.7 mL 1,2,4-trichlorobenzene and 0.3 mL n-octanol was heated to reflux at 230 °C under a stream of argon, and solid dysprosium (III) acetylacetonate hydrate Dy(acac)_3_·*n*H_2_O (18.0 mg, 39.0 μmol) was added. After 8 min, the reaction mixture was cooled to ambient temperature. The reaction mixture was transferred to a chromatographic column packed with alumina in a mixture of CHCl_3_ and hexane (1:1 *v*/*v*). The target complex was isolated by elution with a CHCl_3_-hexane mixture (4:1 *v*/*v*), followed by a mixture of CHCl_3_ with 0→1% MeOH as a dark-blue solid (21.2 mg, yield 57%).

MALDI TOF: *m*/*z* calculated for C_192_H_232_DyN_24_O_36_Y 3703.5, found 3704.3 [M^+^].

UV-vis (CHCl_3_) λ_max_ (nm) (log ε): 646 (4.95), 546 (4.43), 354 (5.14), 294 (5.10).

UV-vis (Toluene) λ_max_ (nm) (log ε): 698 (4.65), 642 (5.40), 363 (5.25), 293 (5.11).

^1^H-NMR (600 MHz, CDCl_3_) δ −29.74 (br s, 16H, bH_Pc_^i^ + cH_Pc_^o^), −13.94 (br s, 8H, 1^ib^), −12.05 (br s, 8H, α^o^), -8.73 (br s, 8H, 1^ic^), −6.83 (br s, 8H, α^o^′), −6.68 (m, 16H, 2^ib,ic^), −5.45 (m, 16H, 3^ib,ic^), −4.31 (s, 8H, β^o^), −4.13 (s, 24H, CH_3_^i^), −2.86 (s, 8H, β^o^′), −1.04 (m, 8H, γ^o^), −0.21 (m, 16H, γ^o^′ + δ^o^), 0.05 (s, 24H, CH_3_^o^), 0.53 (br m, 32H, δ^o^′ + 2^o^ + 3^o,o^′), 1.03 (m, 2^o^′), 2.19 (d, 25 Hz, 8H, 1^o^), 6.41 (d, 25 Hz, 8H, 1^o^′), 16.55 (br s, 8H, bH_Pc_^o^).

^1^H-NMR (600 MHz, Toluene-*d*_8_) δ −28.26 (br s, 8H, bH_Pc_^i^), −22.88 (br, 8H, cH_Pc_^o^), −16.45 (br s, 8H, 1^ib^), −10.96 (br s, 8H, α^o^), −9.68 (br s, 8H, 1^ic^), −7.17 and −6.89 (2s, 2×8H, 2^ib^ and 2^ic^), −6.61 and −6.46 (2s, 2×8H, 3^ib^ and 3^ic^), −4.86 (s, 32H, CH_3_^i^ + α^o^′), −3.56 (s, 8H, β^o^), −2.13 (s, 8H, β^o^′), −0.52 (s, 8H, γ^o^), 0.25 (d, 8H, γ^o^′), 0.41 (s, 24H, CH_3_^o^), 0.56 (s, 8H, δ^o^), 0.83 (s, 8H, δ^o^′), 0.92 and 0.98 (2s, 2×8H, 3^o,o^′), 1.08 and 1.14 (2s, 2×8H, 2^o,o^′), 2.75 (d, 29 Hz, 8H, 1^o^), 6.65 (d, 29 Hz, 8H, 1^o^′), 17.15 (br s, 8H, bH_Pc_^o^).

***Trisphthalocyaninate [B_4_]Dy[B_4_]Dy[C_4_]:*** A solution of phthalocyanines **Dy[B_4_]_2_** (25.5 mg, 10.9 μmol) and **H_2_[C_4_]** (17.4 mg, 13.6 μmol) in a mixture of 2.7 mL 1,2,4-trichlorobenzene and 0.3 mL n-octanol was heated to reflux at 230 °C under a stream of argon, and solid Dy(acac)_3_·*n*H_2_O (18.8 mg, 40.9 μmol) was added. After 8 min, the reaction mixture was cooled to ambient temperature. The reaction mixture was transferred to a chromatographic column packed with alumina in a mixture of CHCl_3_ and hexane (1:1 *v*/*v*). The target complex was isolated by elution with a CHCl_3_-hexane mixture (4:1 *v*/*v*), followed by a mixture of CHCl_3_ with 0→1% MeOH as a dark-blue solid (27 mg, yield 66%).

MALDI TOF: *m*/*z* calculated for C_192_H_232_Dy_2_N_24_O_36_ 3776.6, found *m*/*e*—3777.2 [M^+^].

UV-vis (CHCl_3_) λ_max_ (nm) (log ε): 645 (5.04), 547 (4.53), 355 (5.28), 293 (5.22).

UV-vis (Toluene) λ_max_ (nm) (log ε): 696 (4.65), 642 (5.40), 364 (5.27), 292 (5.13).

^1^H NMR (600 MHz, CDCl_3_) δ −71.54 (br s, 8H, bH_Pc_^i^), −31.56 (br s, 8H, 1^ib^), −30.69 (br s, 8H, 1^ic^), −22.39 (br s, 8H, cH_Pc_^o^), −19.74 (br s, 8H, bH_Pc_^o^), −18.64 and −18.29 (2s, 2×8H, 2^ib^ and 2^ic^), −15.95 and −15.75 (2s, 2×8H, 3^ib^ and 3^ic^), −15.43 (br s, 8H, 1^o^), −14.05 (br s, 8H, α^o^), −11.86 (s, 24H, CH_3_^i^), −7.87 and −7.25 (2s, 2×8H, 3^o^ and 3^o^′), −6.46 (s, 16H, 3^o,o^′), −6.11 (s, 8H, β^o^), −5.42 (br s, 8H, 1^o^′), −4.89 (s, 24H, CH_3_^o^), −5.49 (br s, 8H, α^o^′), −3.06 (s, 8H, β^o^′), −2.34 (m, 8H, γ^o^), −1.29 (s, 8H, δ^o^), −0.65 (m, 8H, γ^o^′), −0.12 (s, 8H, δ^o^′).

^1^H NMR (600 MHz, Toluene-*d*_8_) δ −64.60 (br s, 8H, bH_Pc_^i^), −30.13 (br s, 8H, 1^ic^), −29.97 (br s, 8H, 1^ib^), −15.96 (s, 16H, 2^ib,ic^), −15.07 (br s, 8H, cH_Pc_^i^), −14.67 (s, 16H, 3^ib,ic^), −14.02 (br s, 8H, bH_Pc_^i^), −12.87 (br s, 8H, 1^o^), −12.39 (br s, 8H, α^o^), −10.84 (s, 24H, CH_3_^i^), −5.75 (m, 16H, 2^o,o^′), −4.88 (s, 8H, β^o^), −4.79 (s, 16H, 3^o,o^′), −3.55 (s, 24H, CH_3_^o^), −2.32 (br s, 8H, 1^o^′), −2.05 (s, 8H, β^o^′), −1.99 (br s, 8H, α^o^′), −1.37 (m, 8H, γ^o^), 0.22 (m, 8H, γ^o^′), 0.92 (s, 16H, δ^o,o^′).

***Trisphthalocyaninate [B_4_]Tb[B_4_]Dy[C_4_]:*** A solution of phthalocyanines **Tb[B_4_]_2_** (27.0 mg, 11.5 μmol) and **H_2_[C_4_]** (18.0 mg, 14.1 μmol) in a mixture of 2.7 mL 1,2,4-trichlorobenzene and 0.3 mL n-octanol was heated to reflux at 230 °C under a stream of argon, and solid Dy(acac)_3_·*n*H_2_O (21.0 mg, 45.7 μmol) was added. After 8 min, the reaction mixture was cooled to ambient temperature. The reaction mixture was transferred to a chromatographic column packed with alumina in a mixture of CHCl_3_ and hexane (1:1 *v*/*v*). The target complex was isolated by elution with a CHCl_3_-hexane mixture (4:1 *v*/*v*), followed by a mixture of CHCl_3_ with 0→1% MeOH as a dark-blue solid (20 mg, yield 45%).

MALDI TOF: *m*/*z* calculated for C_192_H_232_DyN_24_O_36_Tb 3773.6, found 3774.3 [M^+^].

UV-vis (CHCl_3_) λ_max_ (nm) (log ε): 646 (5.07), 545 (4.52), 354 (5.24), 293 (5.19).

UV-vis (Toluene) λ_max_ (nm) (log ε): 695 (4.68), 643 (5.47), 363 (5.26), 293 (5.13).

^1^H NMR (600 MHz, CDCl_3_) δ −118.51 (br s, 8H, bH_Pc_^i^), −60.39 (br s, 8H, bH_Pc_^o^), −57.29 (br s, 8H, 1^ib^), −49.37 (br s, 8H, 1^ic^), −35.40 (br s, 8H, 1^o^), −32.17 and −31.58 (2s, 2×8H, 2^ib^ and 2^ic^), −27.90 and −27.42 (2s, 2×8H, 3^ib^ and 3^ic^), −20.68 (s, 24H, CH_3_^i^), −19.23 (br s, 8H, 1^o^′), −17.55 and −16.65 (2s, 2×8H, 2^o^ and 2^o^′), −16.47 (s, 8H, α^o^), −14.60 (s, 16H, 3^o,o^′), −13.11 (br s, 8H, cH_Pc_^o^), −10.70 (s, 24H, CH_3_^o^), −8.11 (s, 8H, β^o^), −4.02 (d, 8H, γ^o^), −3.27 (s, 8H, δ^o^), −2.77 (s, 8H, β^o^′), −1.99 (br s, 8H, α^o^′), −1.15 (d, 8H, γ^o^′), −0.53 (s, 8H, δ^o^′).

^1^H NMR (600 MHz, Toluene-*d*_8_) δ −97.98 (br s, 8H, bH_Pc_^i^), −48.46 (br s, 8H, 1^ib^), −43.16 (br s, 8H, bH_Pc_^o^), −42.45 (br s, 8H, 1^ic^), −27.43 (br s, 8H, 1^o^), −24.44 and −24.08 (2s, 2×8H, 2^ib^ and 2^ic^), −22.21 and −21.96 (2s, 2×8H, 3^ib^ and 3^ic^), −16.24 (s, 24H, CH_3_^i^), −13.35 (br s, 8H, α^o^), −12.18 and −12.04 (2s, 2×8H, 2^o^ and 2^o^′), −10.98 (br s, 8H, 1^o^′), −10.19 (s, 16H, 3^o,o^′), −7.28 (s, 24H, CH_3_^o^), −5.94 (s, 8H, β^o^), −2.08 (m, 8H, γ^o^), −1.86 (s, 8H, β^o^′), 0.00 (s, 8H, δ^o^), 0.20 (m, 8H, γ^o^′), 0.93 (br s, 8H, α^o^′), 1.06 (s, 8H, δ^o^′).

***Trisphthalocyaninate [B_4_]Dy[B_4_]Tb[C_4_]:*** A solution of phthalocyanines **Dy[B_4_]_2_** (29.0 mg, 12.4 μmol) and **H_2_[C_4_]** (19.7 mg, 15.5 μmol) in a mixture of 2.7 mL 1,2,4-trichlorobenzene and 0.3 mL n-octanol was heated to reflux at 230 °C under a stream of argon, and solid terbium (III) acetylacetonate hydrate Tb(acac)_3_·*n*H_2_O (22.0 mg, 46.5 μmol) was added. After 10 min, the reaction mixture was cooled to ambient temperature. The reaction mixture was transferred to a chromatographic column packed with alumina in a mixture of CHCl_3_ and hexane (1:1 *v*/*v*). The target complex was isolated by elution with a CHCl_3_-hexane mixture (4:1 *v*/*v*), followed by a mixture of CHCl_3_ with 0→1% MeOH as a dark-blue solid (29 mg, yield 62%).

MALDI TOF: *m*/*z* calculated for C_192_H_232_DyN_24_O_36_Tb 3773.6, found 3774.3 [M^+^].

UV-vis (CHCl_3_) λ_max_ (nm) (log ε): 645 (5.00), 550 (4.46), 354 (5.18), 293 (5.14).

UV-vis (Toluene) λ_max_ (nm) (log ε): 695 (4.61), 643 (5.39), 363 (5.20), 294 (5.07).

^1^H NMR (600 MHz, CDCl_3_) δ −107.81 (br s, 8H, bH_Pc_^i^), −57.71 (br s, 8H, cH_Pc_^i^), −48.90 (br s, 8H, 1^ic^), −44.48 (br s, 8H, 1^ib^), −30.28 (br s, 8H, α^o^), −27.57 and −27.15 (2s, 2×8H, 2^ib^ and 2^ic^), −23.25 (m, 16H, 3^ib,ic^), −17.83 (br s, 8H, 1^o^), −17.32 (s, 24H, CH_3_^i^), −15.56 (br s, 8H, α^o^′), −14.33 (s, 8H, β^o^), −11.63 (br s, 8H, bH_Pc_^o^), −9.78 (s, 8H, β^o^′), −9.38 and −8.51 (2s, 2×8H, 2^o^ and 2^o^′), −7.73 (s, 16H, 3^o,o^′), −7.75 (m, 8H, γ^o^), −6.05 (s, 24H, CH_3_^o^), −5.33 (s, 8H, δ^o^), −4.60 (m, 8H, γ^o^′), −3.54 (br s, 8H, 1^o^′), −3.15 (s, 8H, δ^o^′).

^1^H NMR (600 MHz, Toluene-*d*_8_) δ −100.40 (br s, 8H, bH_Pc_^i^), −50.04 (br s, 8H, 1^ic^), −44.57 (br s, 8H, cH_Pc_^o^), −43.19 (br s, 8H, 1^ib^), −26.68 (br s, 8H, α^o^), −24.04 and −24.76 (2s, 2×8H, 2^ib^ and 2^ic^), −22.70 (s, 16H, 3^ib,ic^), −16.69 (s, 24H, CH_3_^i^), −14.12 (br s, 8H, 1^o^), −11.80 (s, 8H, β^o^), −10.39 (br s, 8H, α^o^′), −7.45 (s, 8H, β^o^′), −6.45 and −6.37 (2s, 2×8H, 2^o^ and 2^o^′), −5.58 (m, 8H, γ^o^), −5.40 (s, 16H, 3^o,o^′), −4.17 (s, 32H, bH_Pc_^o^ + CH_3_^o^), −3.07 (m, 8H, γ^o^′), −2.93 (s, 8H, δ^o^), −2.03 (s, 8H, δ^o^′), 0.46 (br s, 8H, 1^o^′).

## Data Availability

Data are contained within this article or the Supplementary Materials.

## Supplementary Materials

The following supporting information can be downloaded at: https://www.mdpi.com/article/10.3390/molecules29020510/s1, Figures S1–S5: MALDI-TOF mass spectra of the synthesized triple-decker complexes; Figure S6: Fragments of X-ray structures of **[C_4_]Yb[C_4_]Y(Pc)·4CHCl_3_·3H_2_O** and **[B_4_]Y[B_4_]Y[C_4_]·10CH_2_Cl_2_**, showing the analogy in localization of solvent molecules and contacts with substituents that stabilize staggered pairwise conformations; Figures S7–S26: ^1^H-NMR and ^1^H-^1^H COSY spectra of the synthesized triple-decker complexes in CDCl_3_ and toluene-*d*_8_.
