# Supplementary material for: Magnetic Anisotropy of Homo- and Heteronuclear Terbium(III) and Dysprosium(III) Trisphthalocyaninates Derived from Paramagnetic 1H-NMR Investigation
Source: Molecules. 2024 Jan 19;29(2):510. doi: 10.3390/molecules29020510 (PMC11154240; doi:10.3390/molecules29020510)
Supplement: Supplementary file 1 [file molecules-29-00510-s001.zip › molecules-2815131-supplementary.pdf]

Magnetic Anisotropy of Homo- and Heteronuclear Terbium (III) and Dysprosium(III) Trisphthalocyaninates Derived from Paramagnetic ^{1}H-NMR Investigation

Ilya D. Kormschikov, Marina A. Polovkova, Gayane A. Kirakosyan [2,3], Alexander G. Martynov [2,*], Yulia G. Gorbunova [2,3] and Aslan Yu. Tsivadze [2,3]

Figure S1. MALDI-TOF mass spectrum of **[B$_4$]Dy[B$_4$]Y[C$_4$]**. 2

Figure S2. MALDI-TOF mass spectrum of **[B$_4$]Y[B$_4$]Dy[C$_4$]**. 2

Figure S3. MALDI-TOF mass spectrum of **[B$_4$]Dy[B$_4$]Dy[C$_4$]**. 3

Figure S4. MALDI-TOF mass spectrum of **[B$_4$]Tb[B$_4$]Dy[C$_4$]**. 3

Figure S5. MALDI-TOF mass spectrum of **[B$_4$]Dy[B$_4$]Tb[C$_4$]**. 4

Figure S6. Fragments of X-ray structures of **[C$_4$]Yb[C$_4$]Y(Pc)·4CHCl$_3$·3H$_2$O** and **[B$_4$]Y[B$_4$]Y[C$_4$]·10CH$_2$Cl$_2$**, showing the analogy in localization of solvent molecules and contacts with substituents which stabilize staggered pairwise conformations. 5

Figure S7. ^{1}H-NMR spectrum of **[B$_4$]Dy[B$_4$]Y[C$_4$]** in CDCl$_3$. 7

Figure S8. ^{1}H-^{1}H COSY spectrum of **[B$_4$]Dy[B$_4$]Y[C$_4$]** in CDCl$_3$. 7

Figure S9. ^{1}H-NMR spectrum of **[B$_4$]Dy[B$_4$]Y[C$_4$]** in toluene-d$_8$. 8

Figure S10. ^{1}H-^{1}H COSY spectrum of **[B$_4$]Dy[B$_4$]Y[C$_4$]** in toluene-d$_8$. 8

Figure S11. ^{1}H-NMR spectrum of **[B$_4$]Y[B$_4$]Dy[C$_4$]** in CDCl$_3$. 9

Figure S12. ^{1}H-^{1}H COSY spectrum of **[B$_4$]Y[B$_4$]Dy[C$_4$]** in CDCl$_3$. 9

Figure S13. ^{1}H-NMR spectrum of **[B$_4$]Y[B$_4$]Dy[C$_4$]** in toluene-d$_8$. 10

Figure S14. ^{1}H-^{1}H COSY spectrum of **[B$_4$]Y[B$_4$]Dy[C$_4$]** in toluene-d$_8$. 10

Figure S15. ^{1}H-NMR spectrum of **[B$_4$]Dy[B$_4$]Dy[C$_4$]** in CDCl$_3$. 11

Figure S16. ^{1}H-^{1}H COSY spectrum of **[B$_4$]Dy[B$_4$]Dy[C$_4$]** in CDCl$_3$. 11

Figure S17. ^{1}H-NMR spectrum of **[B$_4$]Dy[B$_4$]Dy[C$_4$]** in toluene-d$_8$. 12

Figure S18. ^{1}H-^{1}H COSY spectrum of **[B$_4$]Dy[B$_4$]Dy[C$_4$]** in toluene-d$_8$. 12

Figure S19. ^{1}H-NMR spectrum of **[B$_4$]Tb[B$_4$]Dy[C$_4$]** in CDCl$_3$. 13

Figure S20. ^{1}H-^{1}H COSY spectrum of **[B$_4$]Tb[B$_4$]Dy[C$_4$]** in CDCl$_3$. 13

Figure S21. ^{1}H-NMR spectrum of **[B$_4$]Tb[B$_4$]Dy[C$_4$]** in toluene-d$_8$. 14

Figure S22. ^{1}H-^{1}H COSY spectrum of **[B$_4$]Tb[B$_4$]Dy[C$_4$]** in toluene-d$_8$. 14

Figure S23. ^{1}H-NMR spectrum of **[B$_4$]Dy[B$_4$]Tb[C$_4$]** in CDCl$_3$. 15

Figure S24. ^{1}H-^{1}H COSY spectrum of **[B$_4$]Dy[B$_4$]Tb[C$_4$]** in CDCl$_3$. 15

Figure S25. ^{1}H-NMR spectrum of **[B$_4$]Dy[B$_4$]Tb[C$_4$]** in toluene-d$_8$. 16

Figure S26. ^{1}H-^{1}H COSY spectrum of **[B$_4$]Dy[B$_4$]Tb[C$_4$]** in toluene-d$_8$. 16

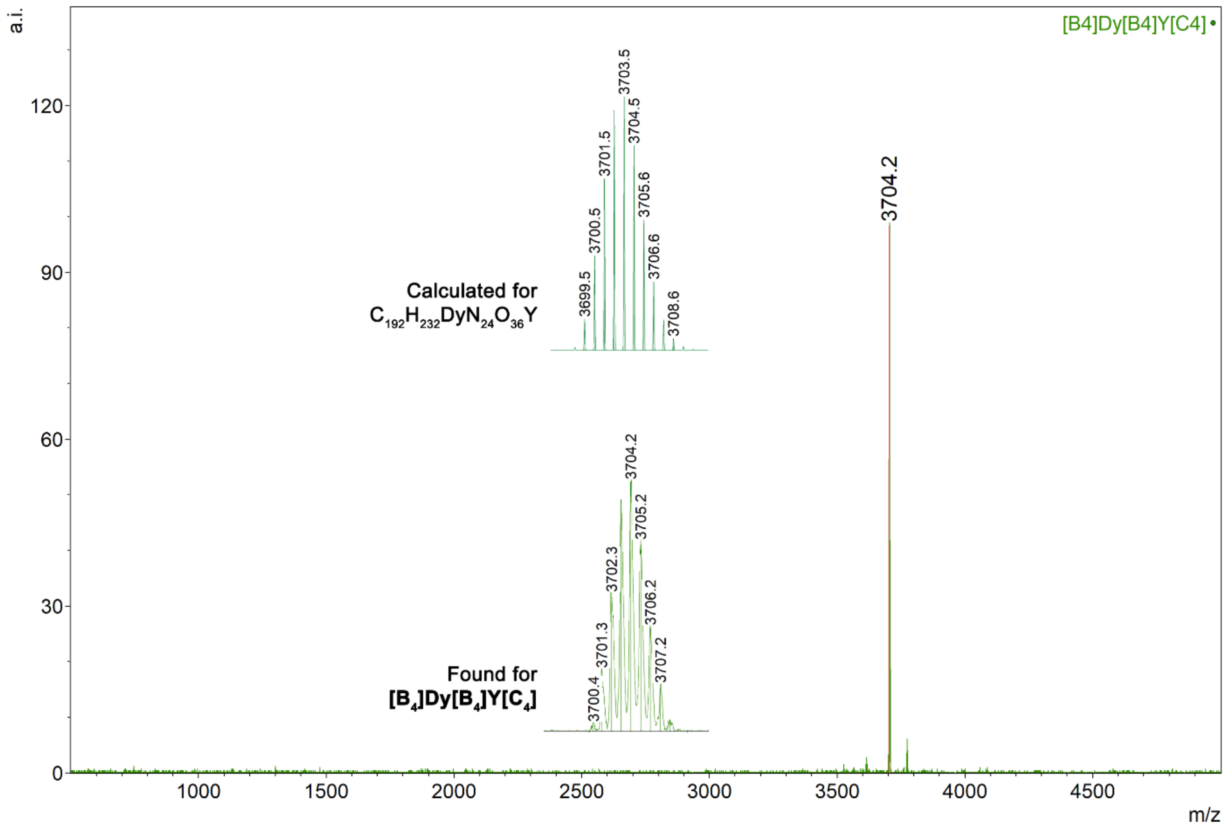

Figure S1. MALDI-TOF mass spectrum of **[B₄]Dy[B₄]Y[C₄]**.

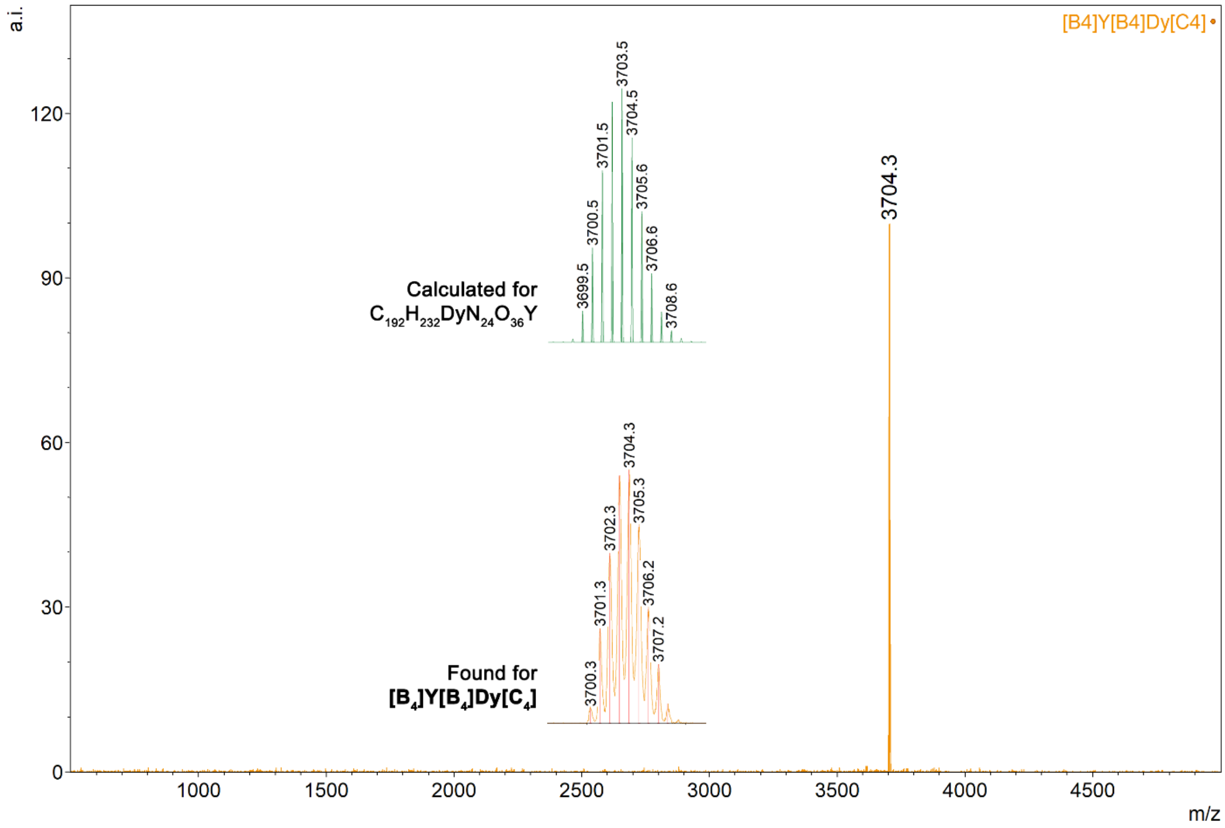

Figure S2. MALDI-TOF mass spectrum of **[B₄]Y[B₄]Dy[C₄]**.

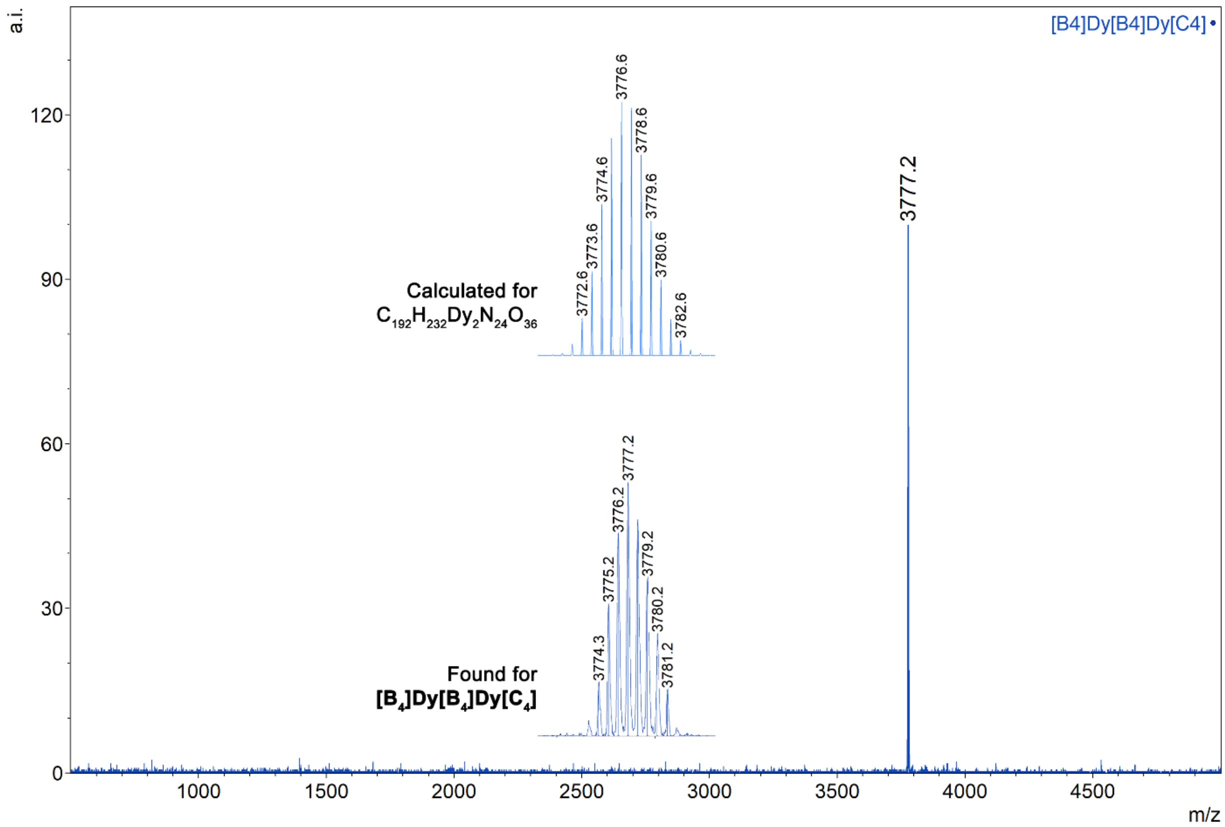

Figure S3. MALDI-TOF mass spectrum of **$[B_4]Dy[B_4]Dy[C_4]$**.

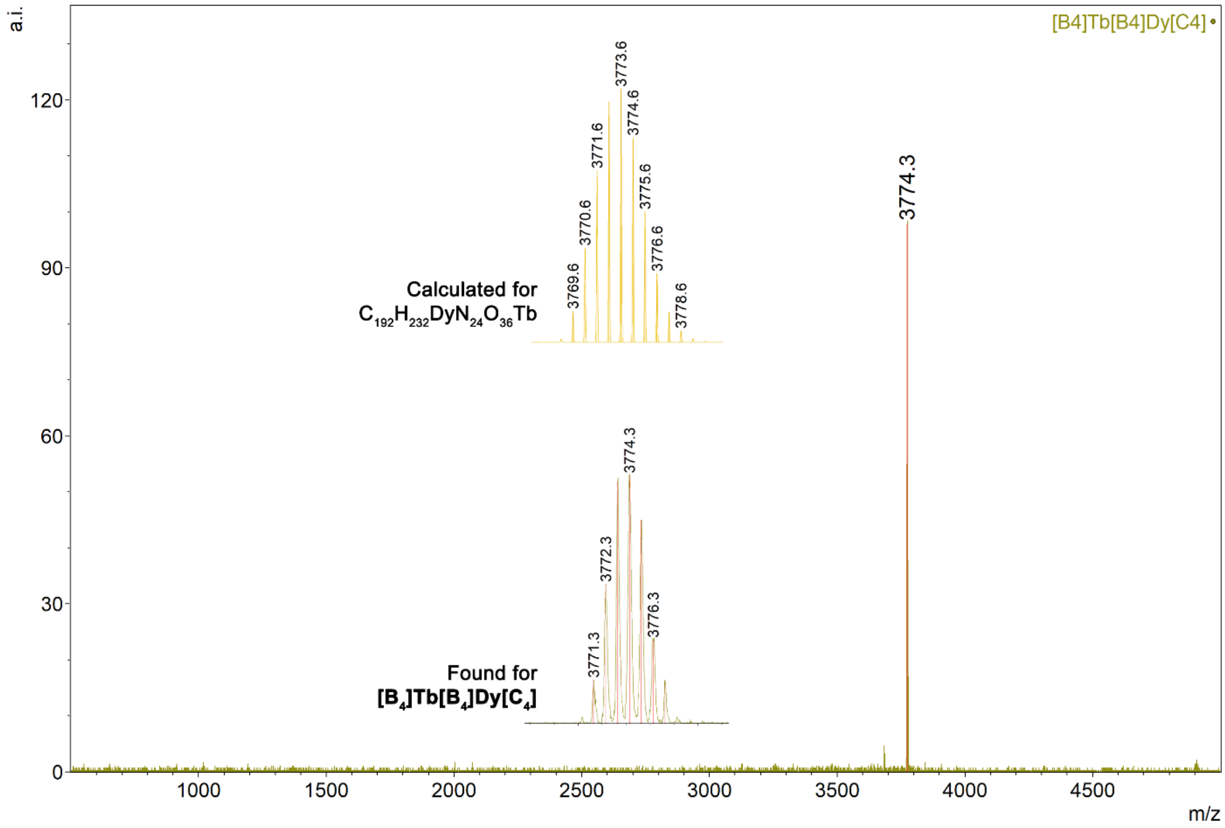

Figure S4. MALDI-TOF mass spectrum of **$[B_4]Tb[B_4]Dy[C_4]$**.

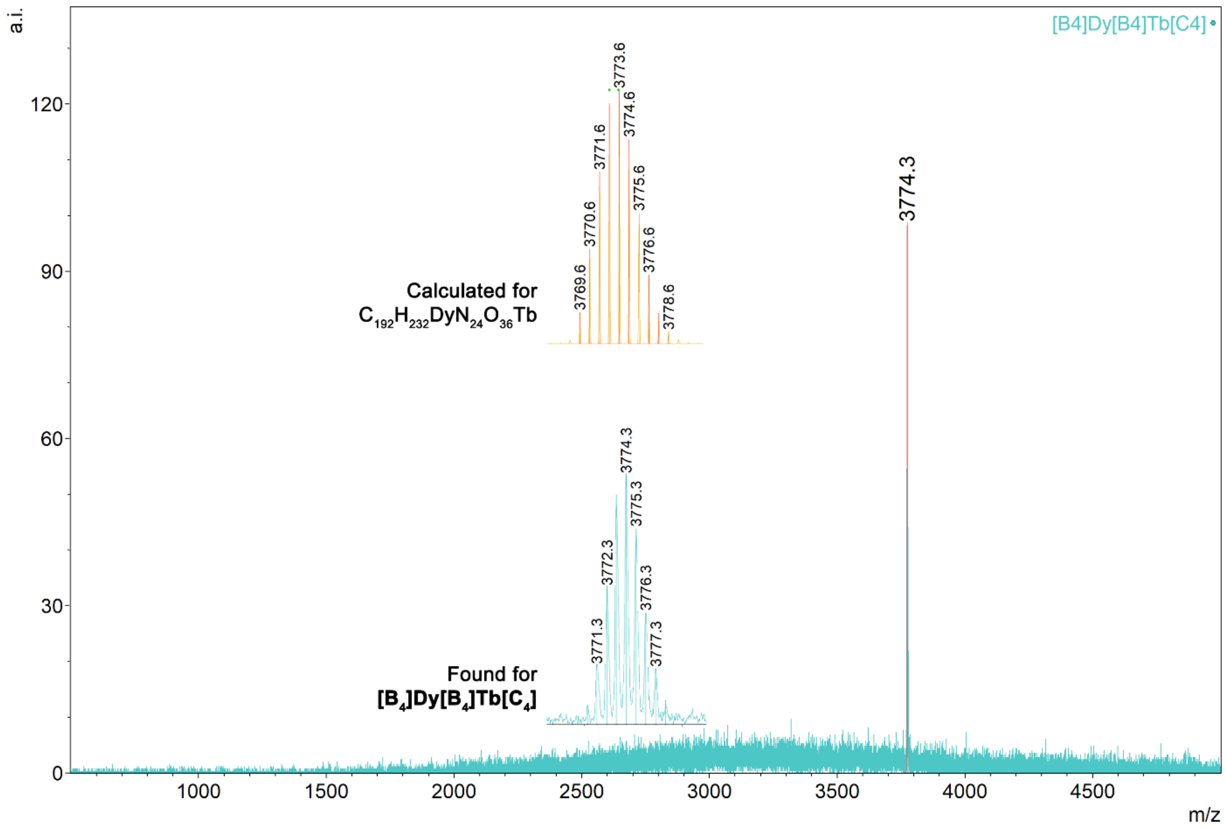

Figure S5. MALDI-TOF mass spectrum of **[B4]Dy[B4]Tb[C4]**.

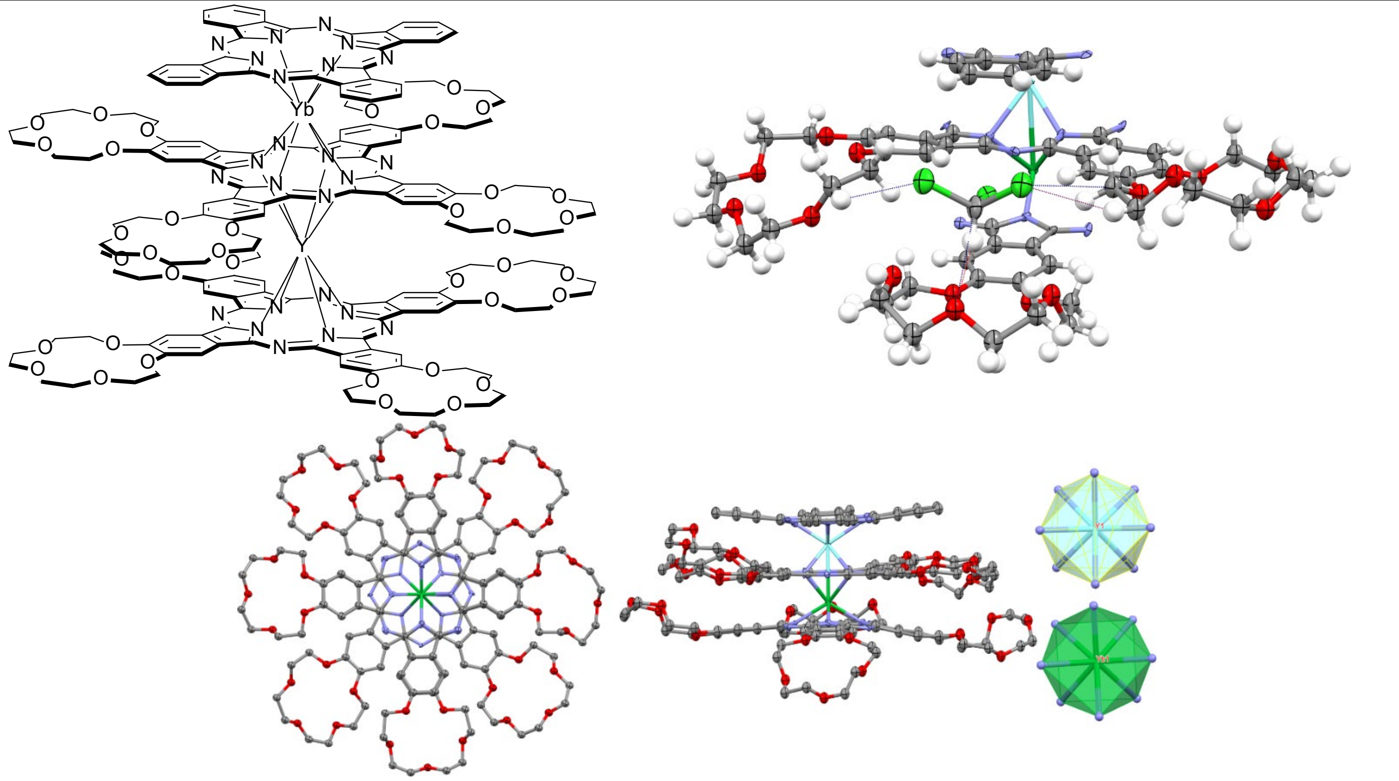

[C_4]Yb[C_4]Y(Pc)·4$CHCl_3$·3H_2O, X-ray
(CCDC EXAXOS, [1])

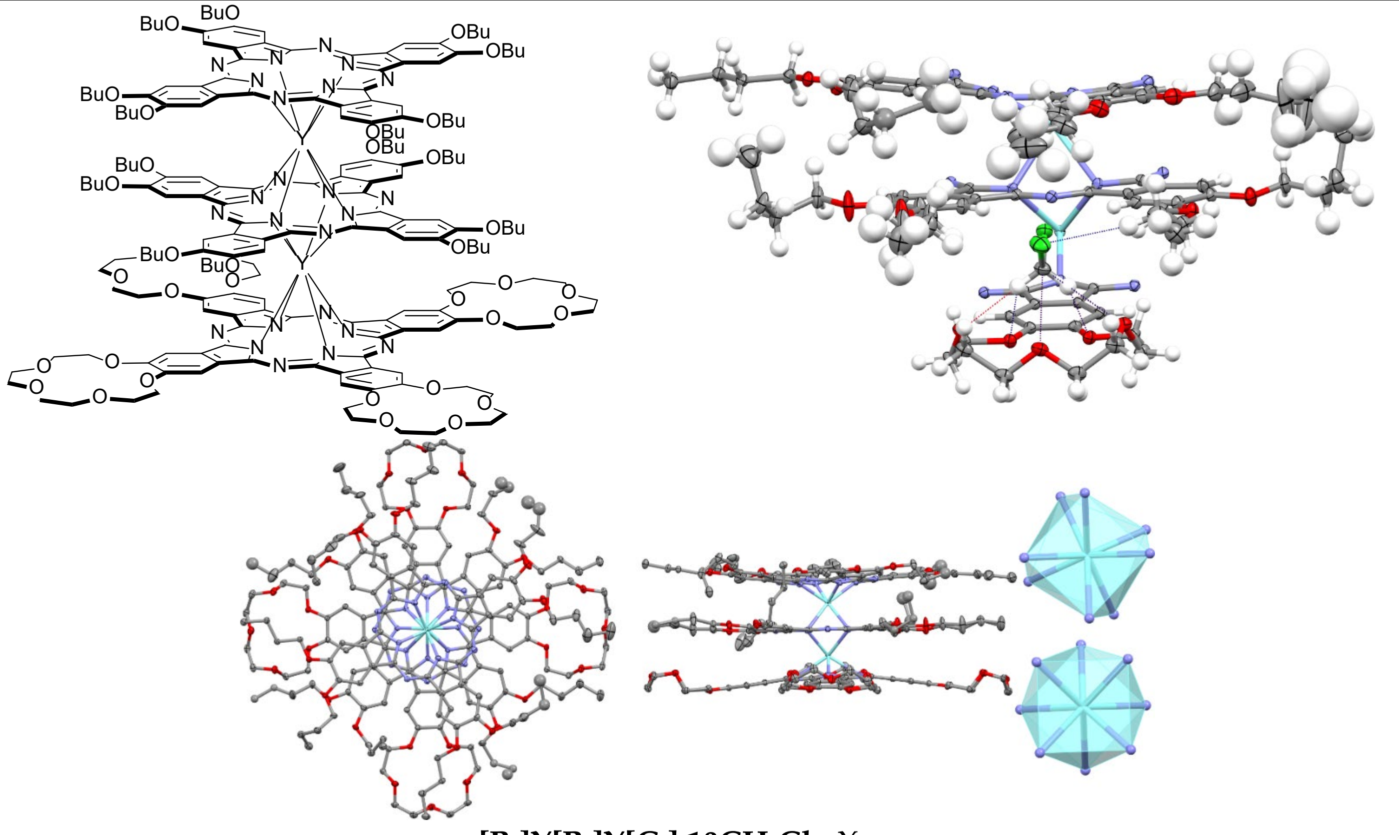

[B_4]Y[B_4]Y[C_4]·10CH_2Cl_2, X-ray
(CCDC FIJTEB [2])

Figure S6. Fragments of X-ray structures of **[C_4]Yb[C_4]Y(Pc)·4$CHCl_3$·3H_2O** and **[B_4]Y[B_4]Y[C_4]·10CH_2Cl_2**, showing the analogy in localization of solvent molecules and contacts with substituents which stabilize staggered pairwise conformations.

In both cases chlorinated solvents are sitting atop the crown-ether rings forming weak CH…O contacts with oxygen atoms of crown-ether rings and CH…Cl contacts with the substituents of the neighboring ligands which stabilizes the pairwise staggered conformation.

1. Polovkova, M.A.; Martynov, A.G.; Birin, K.P.; Nefedov, S.E.; Gorbunova, Y.G.; Tsivadze, A.Y. Determination of the Structural Parameters of Heteronuclear (Phthalocyaninato)Bis(Crownphthalocyaninato)Lanthanide(III) Triple-Deckers in Solution by Simultaneous Analysis of NMR and Single-Crystal X-Ray Data. *Inorg. Chem.* **2016**, *55*, 9258–9269, doi:10.1021/acs.inorgchem.6b01292.

2. Martynov, A.G.; Sinelshchikova, A.A.; Dorovatovskii, P. V.; Polovkova, M.A.; Ovchenkova, A.E.; Birin, K.P.; Kirakosyan, G.A.; Gorbunova, Y.G.; Tsivadze, A.Y. Solvation-Induced Conformational Switching of Trisphthalocyanates for Control of Their Magnetic Properties. *Inorg. Chem.* **2023**, *62*, 10329–10342, doi:10.1021/acs.inorgchem.3c01169.

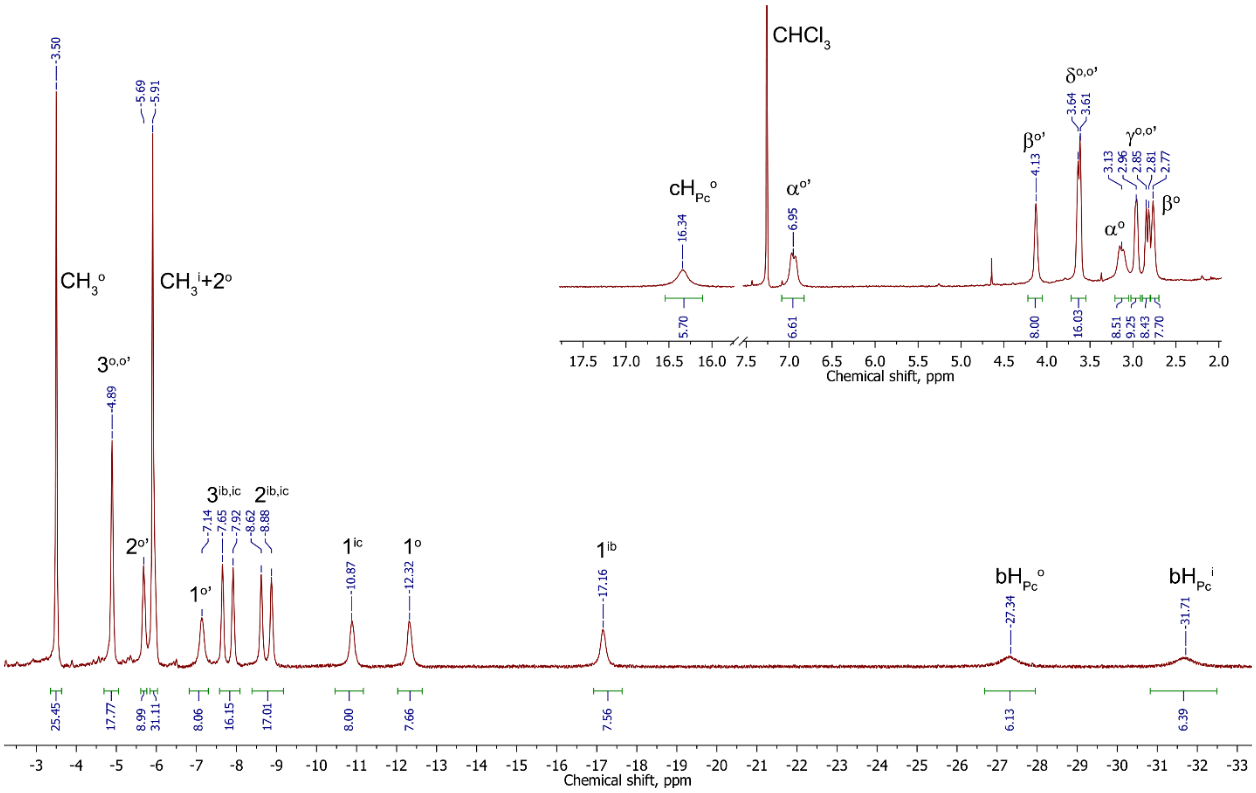

Figure S7. ^{1}H-NMR spectrum of **[B$_4$]Dy[B$_4$]Y[C$_4$]** in CDCl$_3$.

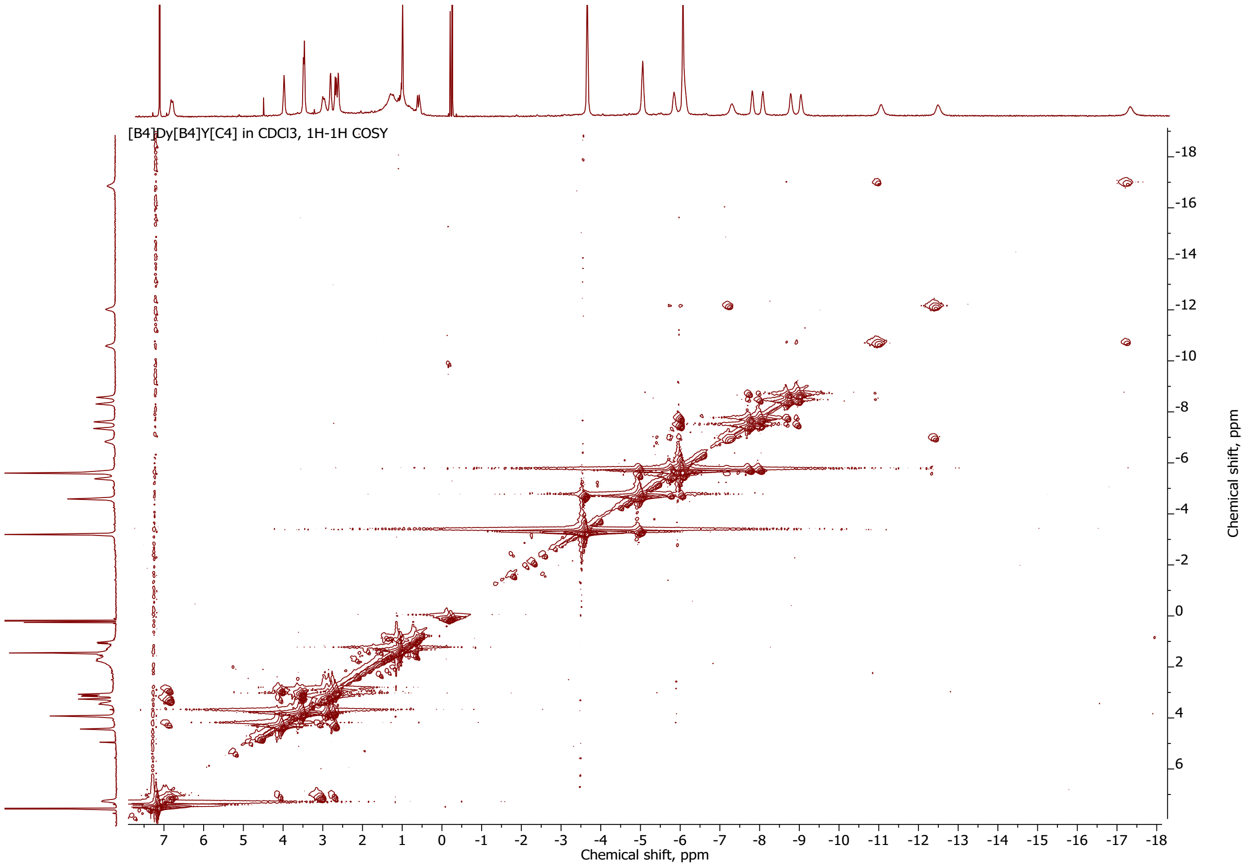

Figure S8. ^{1}H-^{1}H COSY spectrum of **[B$_4$]Dy[B$_4$]Y[C$_4$]** in CDCl$_3$.

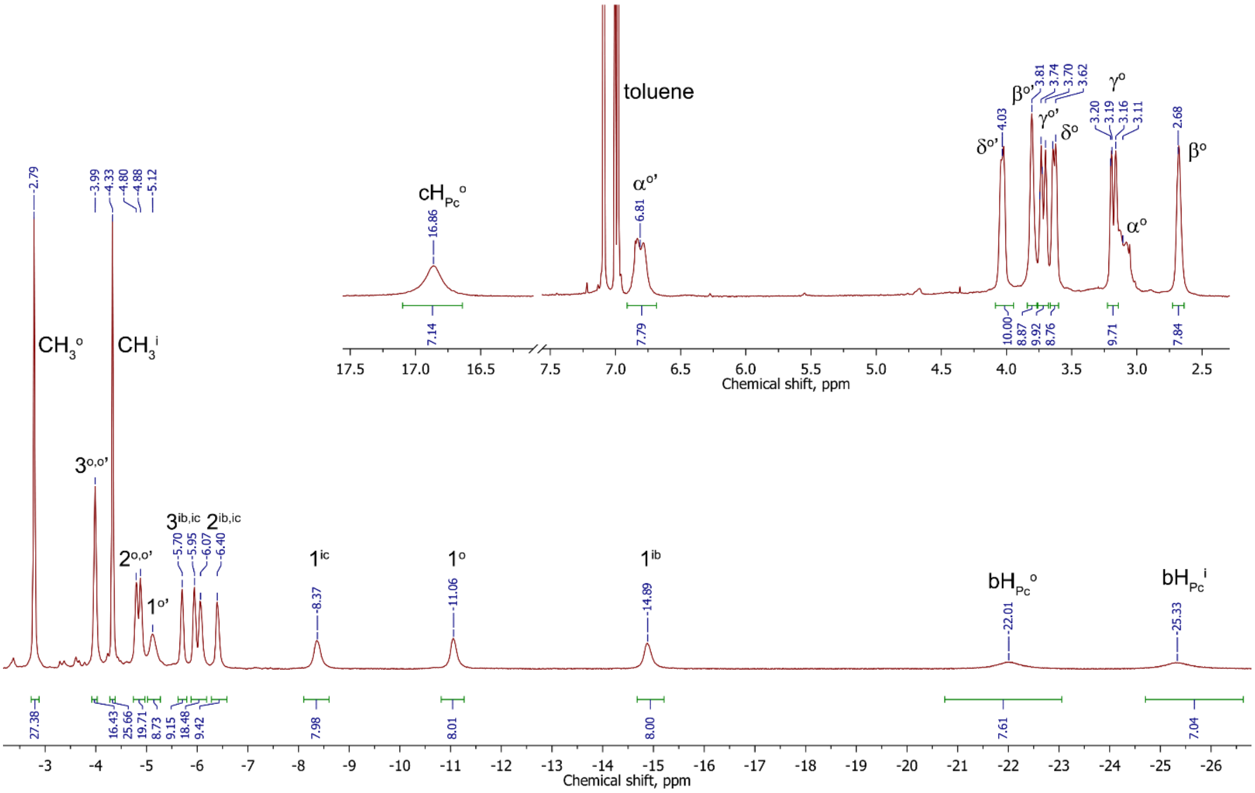

Figure S9. ^{1}H-NMR spectrum of **[B$_4$]Dy[B$_4$]Y[C$_4$]** in toluene-d$_8$.

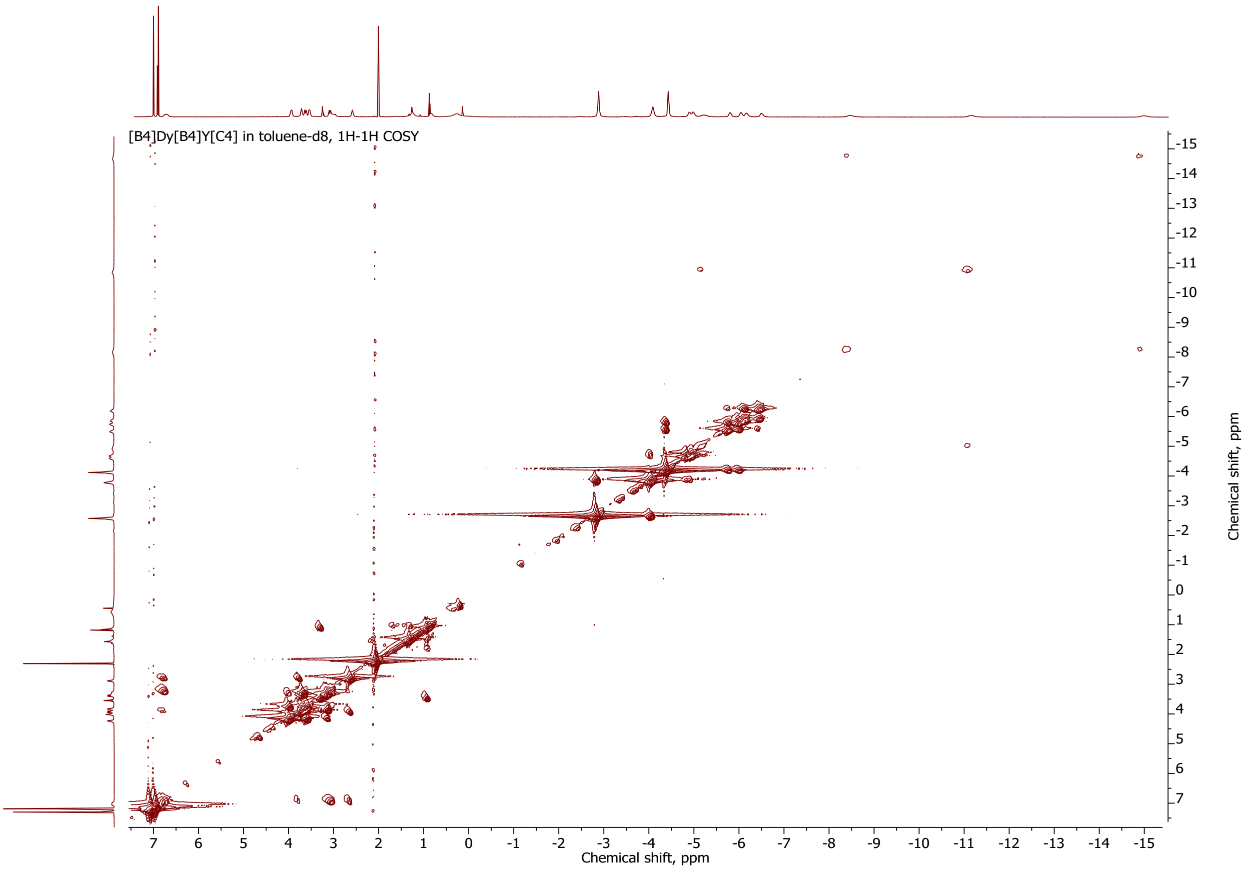

Figure S10. ^{1}H-^{1}H COSY spectrum of **[B$_4$]Dy[B$_4$]Y[C$_4$]** in toluene-d$_8$.

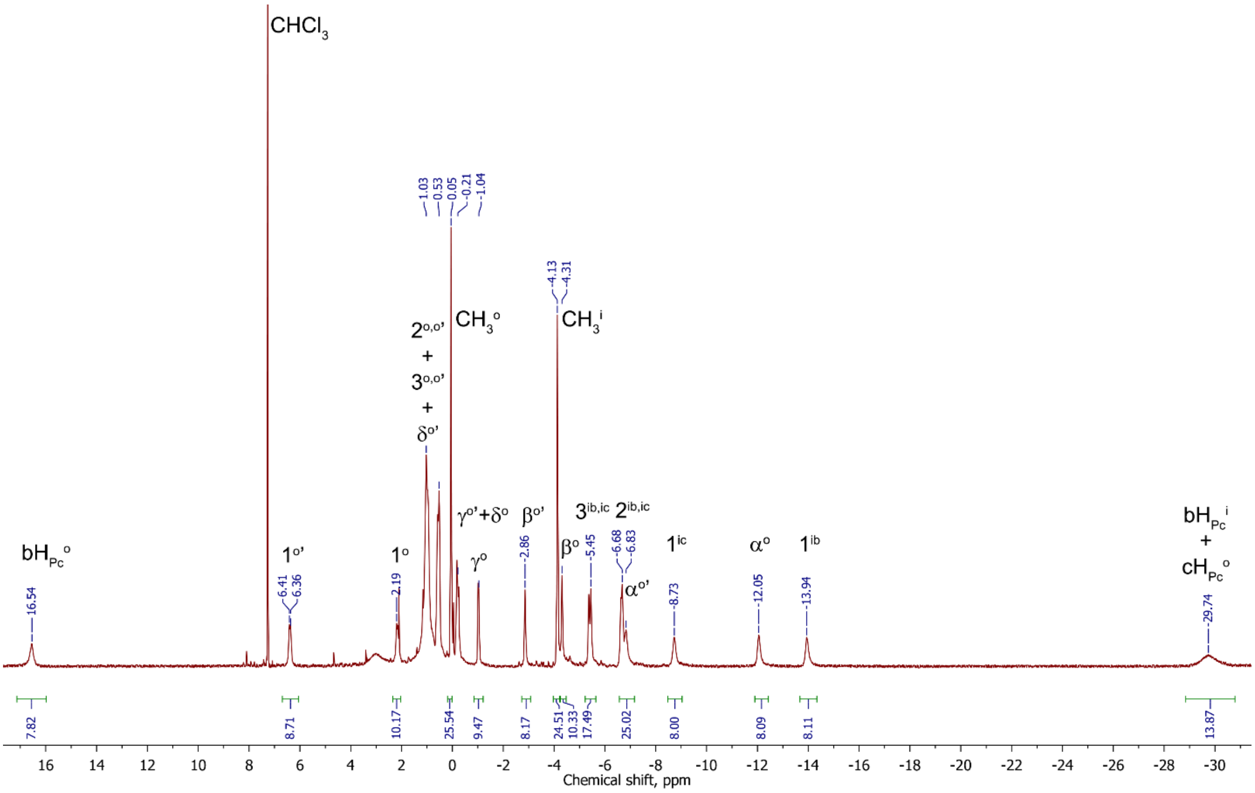

Figure S11. ^{1}H-NMR spectrum of **[B$_4$]Y[B$_4$]Dy[C$_4$]** in $CDCl_3$.

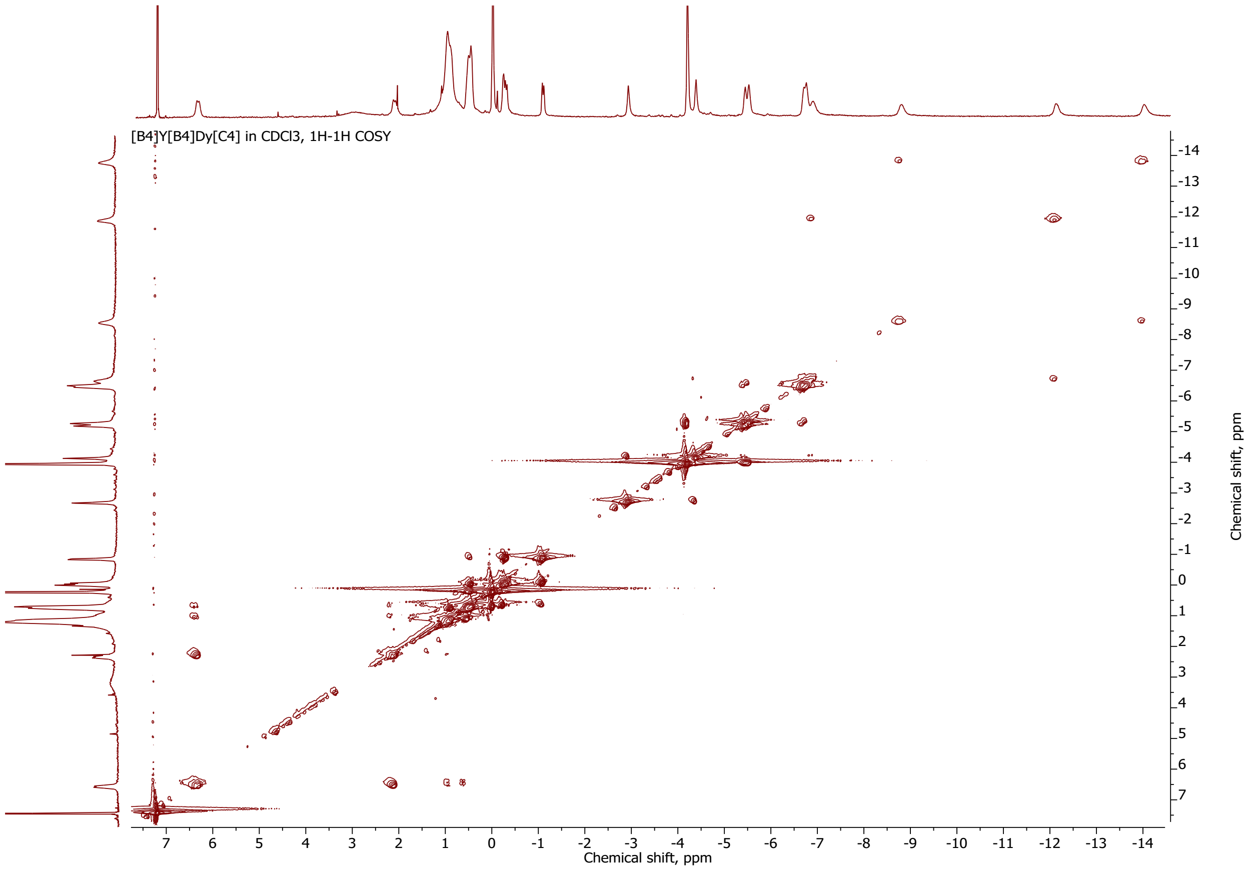

Figure S12. ^{1}H-^{1}H COSY spectrum of **[B$_4$]Y[B$_4$]Dy[C$_4$]** in $CDCl_3$.

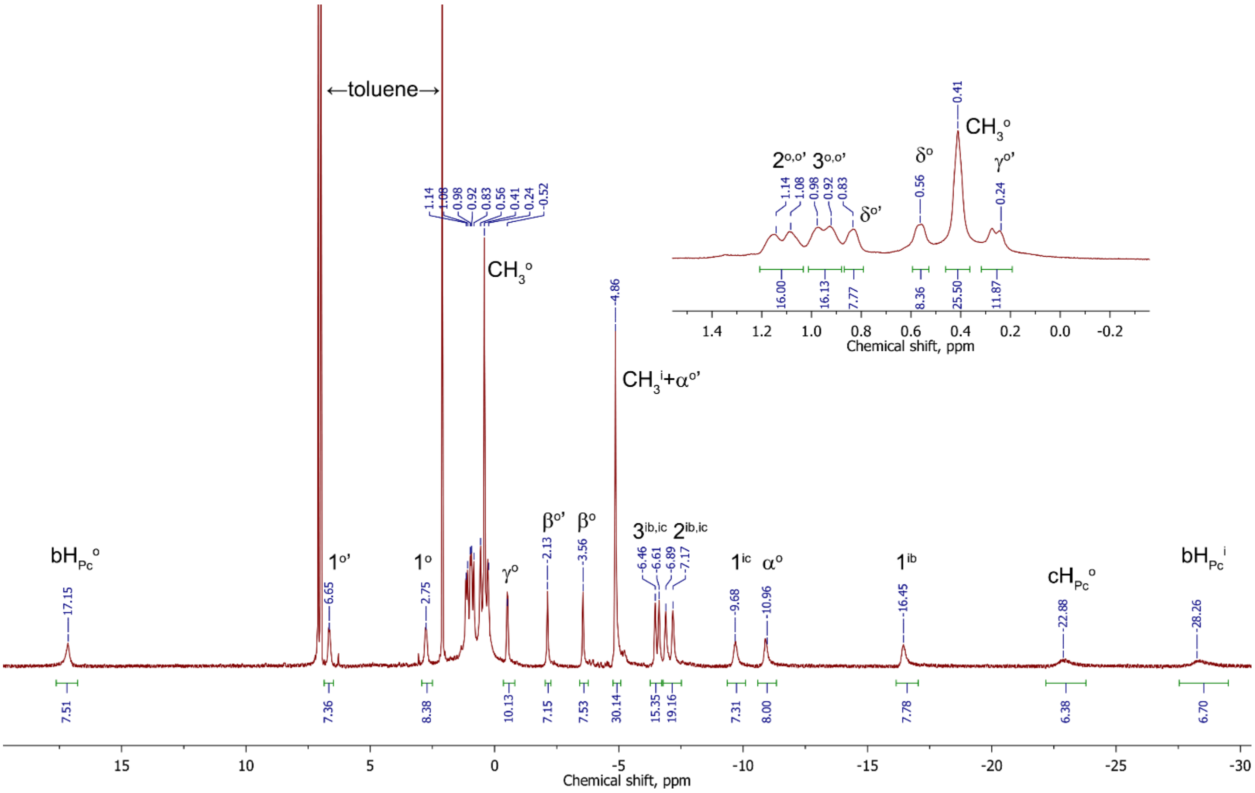

Figure S13. ^{1}H-NMR spectrum of **[B$_4$]Y[B$_4$]Dy[C$_4$]** in toluene-d$_8$.

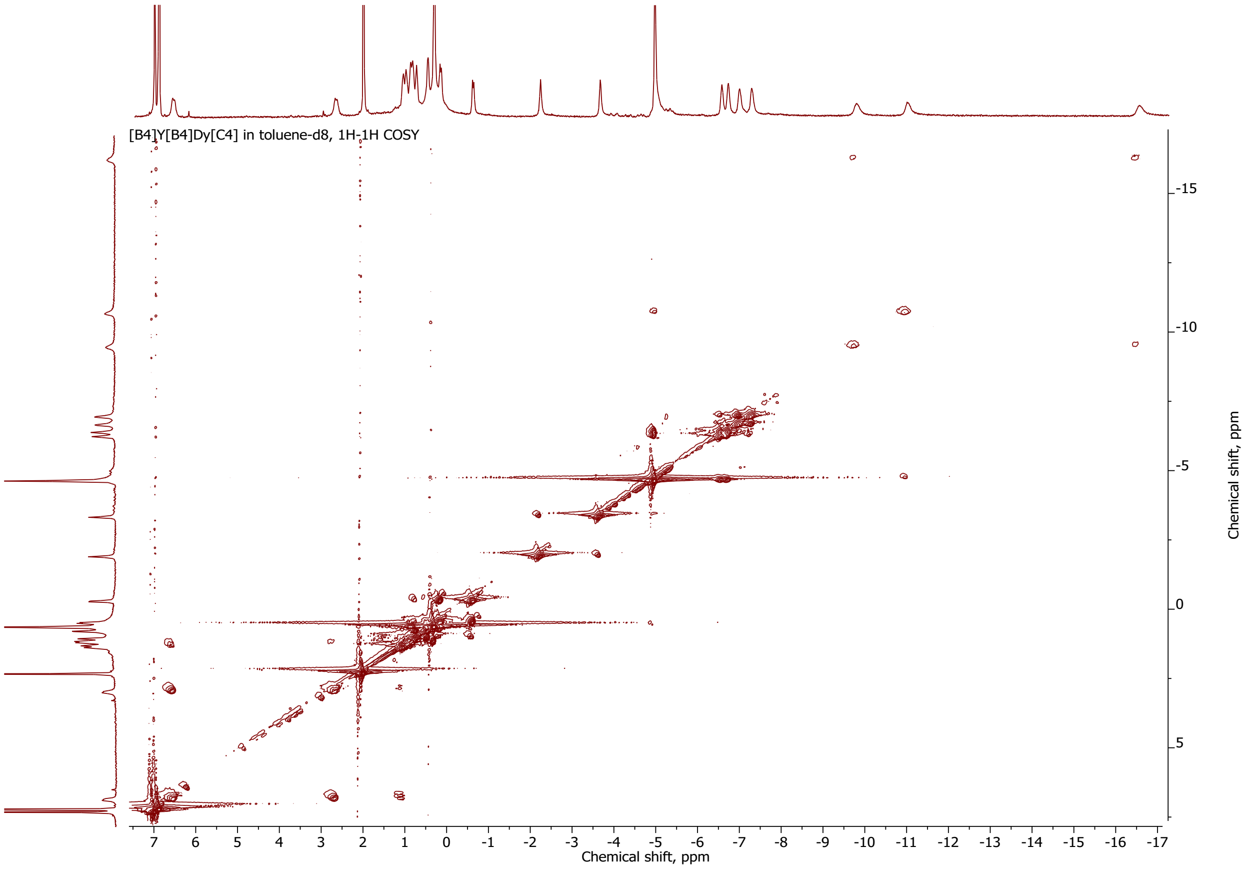

Figure S14. ^{1}H-^{1}H COSY spectrum of **[B$_4$]Y[B$_4$]Dy[C$_4$]** in toluene-d$_8$.

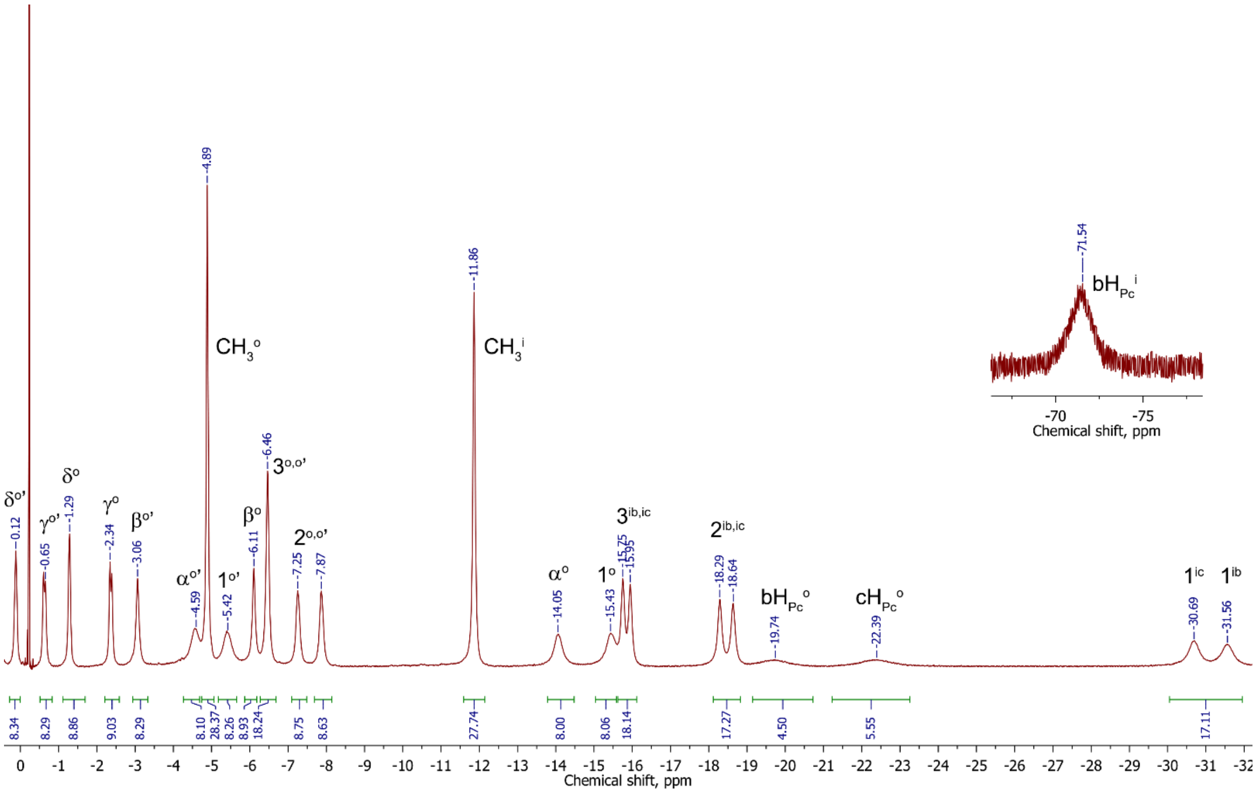

Figure S15. ^{1}H-NMR spectrum of **[B4]Dy[B4]Dy[C4]** in $CDCl_3$.

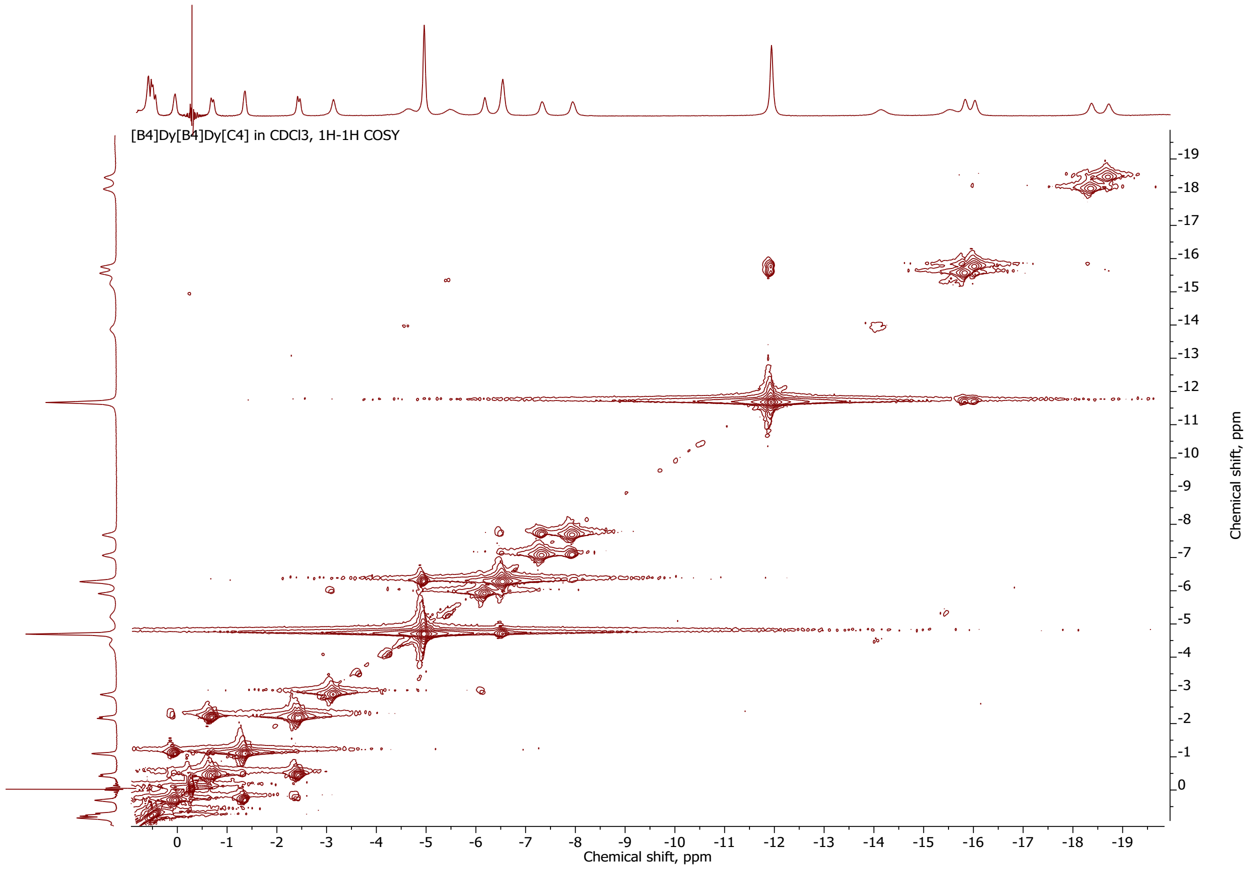

Figure S16. ^{1}H-^{1}H COSY spectrum of **[B4]Dy[B4]Dy[C4]** in $CDCl_3$.

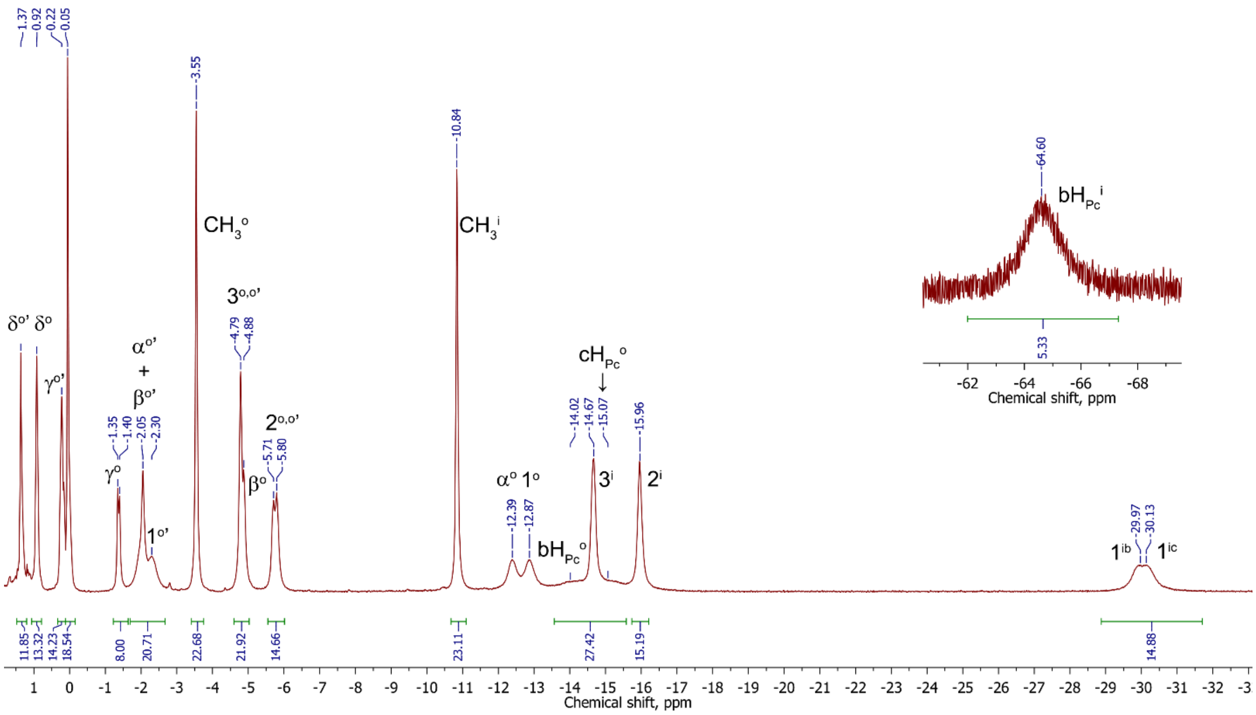

Figure S17. ^{1}H-NMR spectrum of **[B$_4$]Dy[B$_4$]Dy[C$_4$]** in toluene-d$_8$.

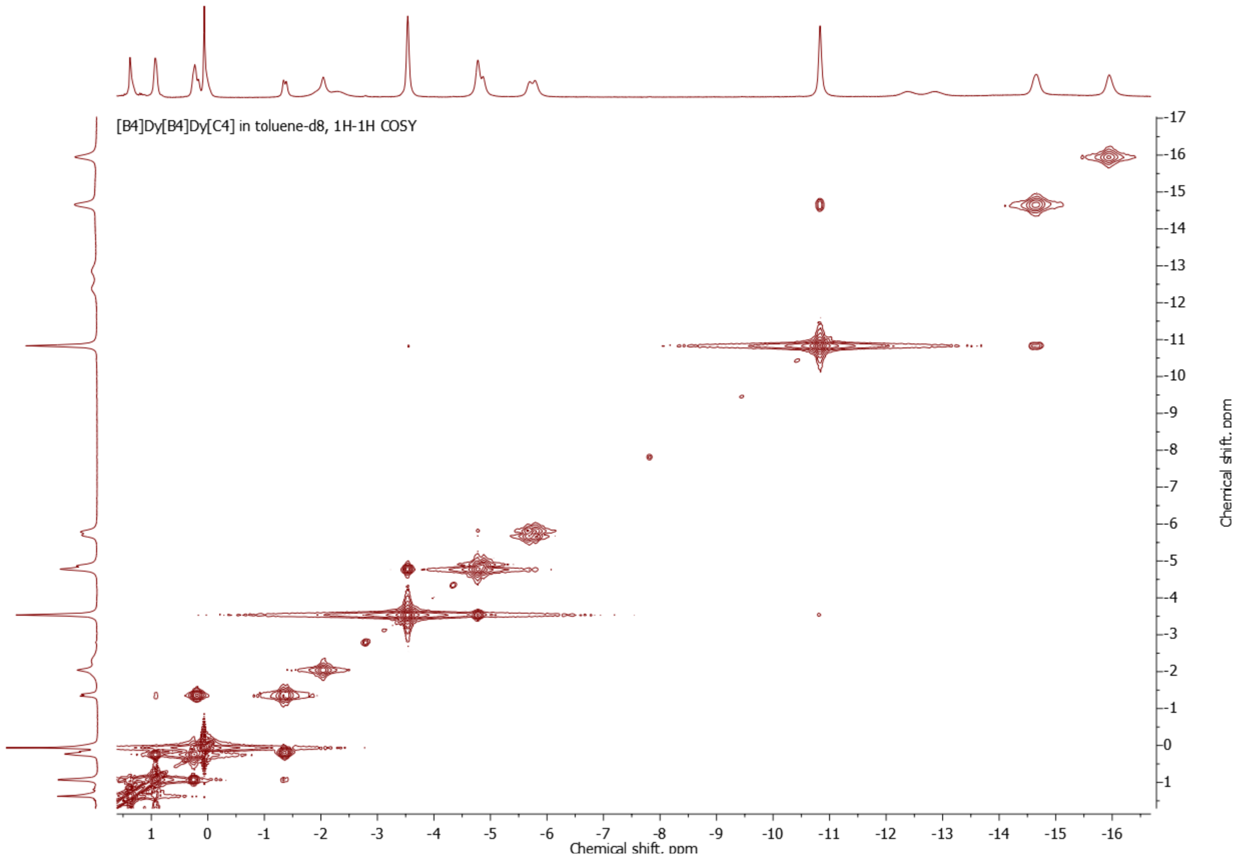

Figure S18. ^{1}H-^{1}H COSY spectrum of **[B$_4$]Dy[B$_4$]Dy[C$_4$]** in toluene-d$_8$.

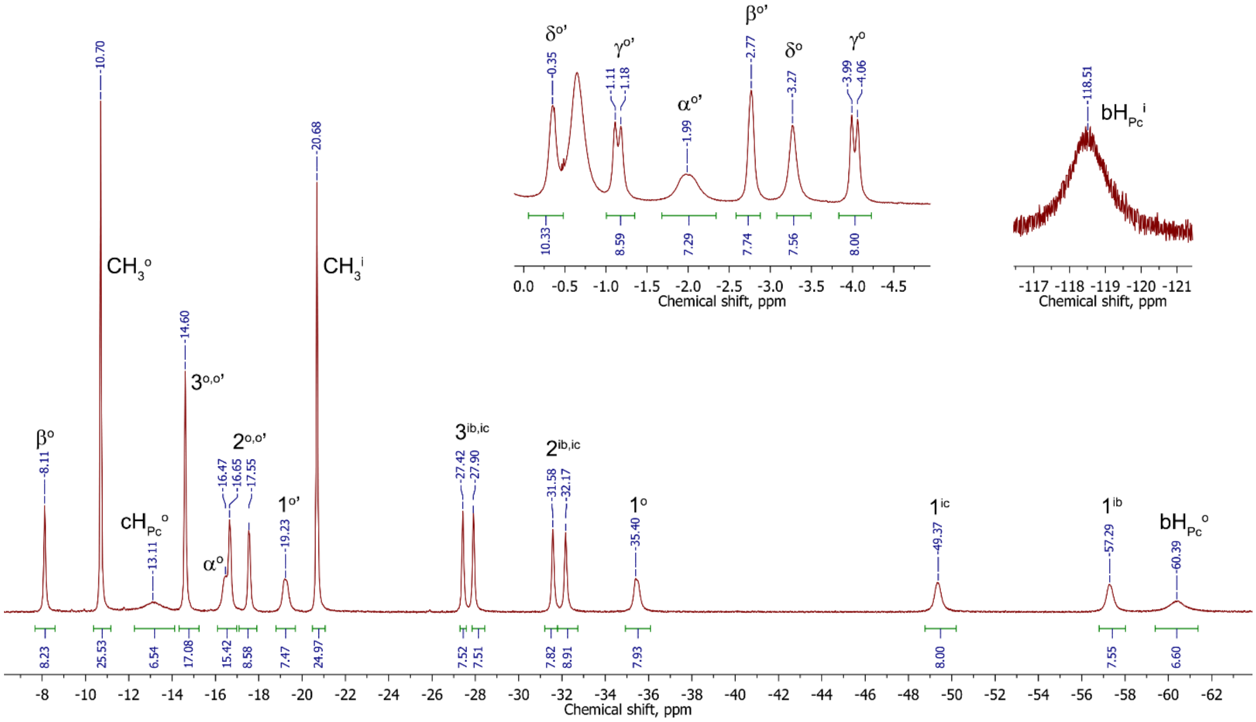

Figure S19. ^{1}H-NMR spectrum of **[B$_4$]Tb[B$_4$]Dy[C$_4$]** in $CDCl_3$.

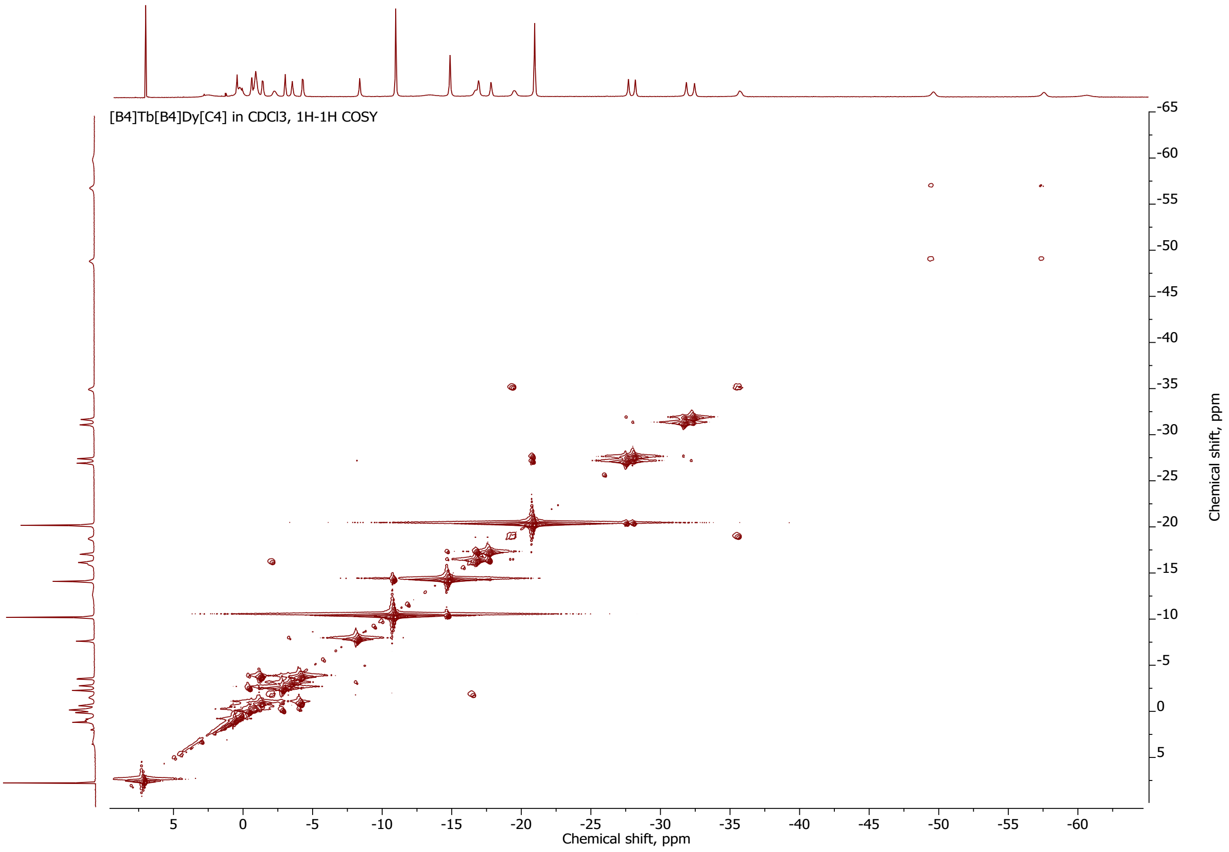

Figure S20. ^{1}H-^{1}H COSY spectrum of **[B$_4$]Tb[B$_4$]Dy[C$_4$]** in $CDCl_3$.

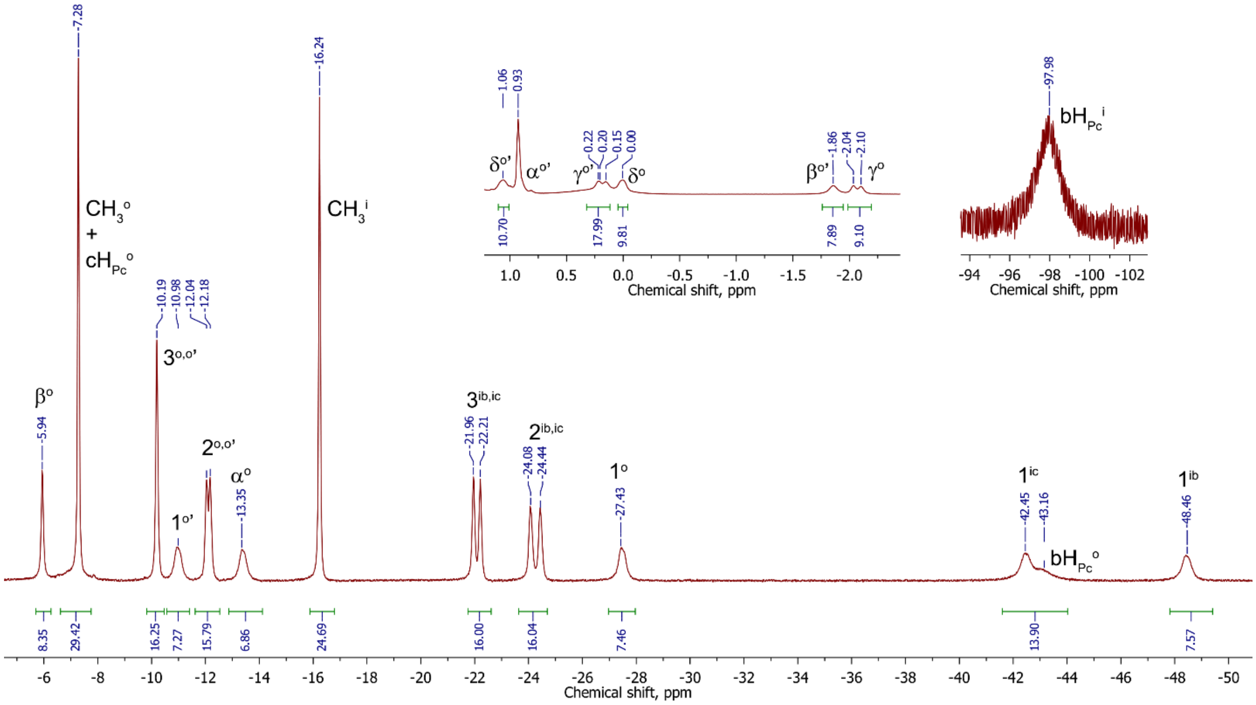

Figure S21. ^{1}H-NMR spectrum of **[B$_4$]Tb[B$_4$]Dy[C$_4$]** in toluene-d$_8$.

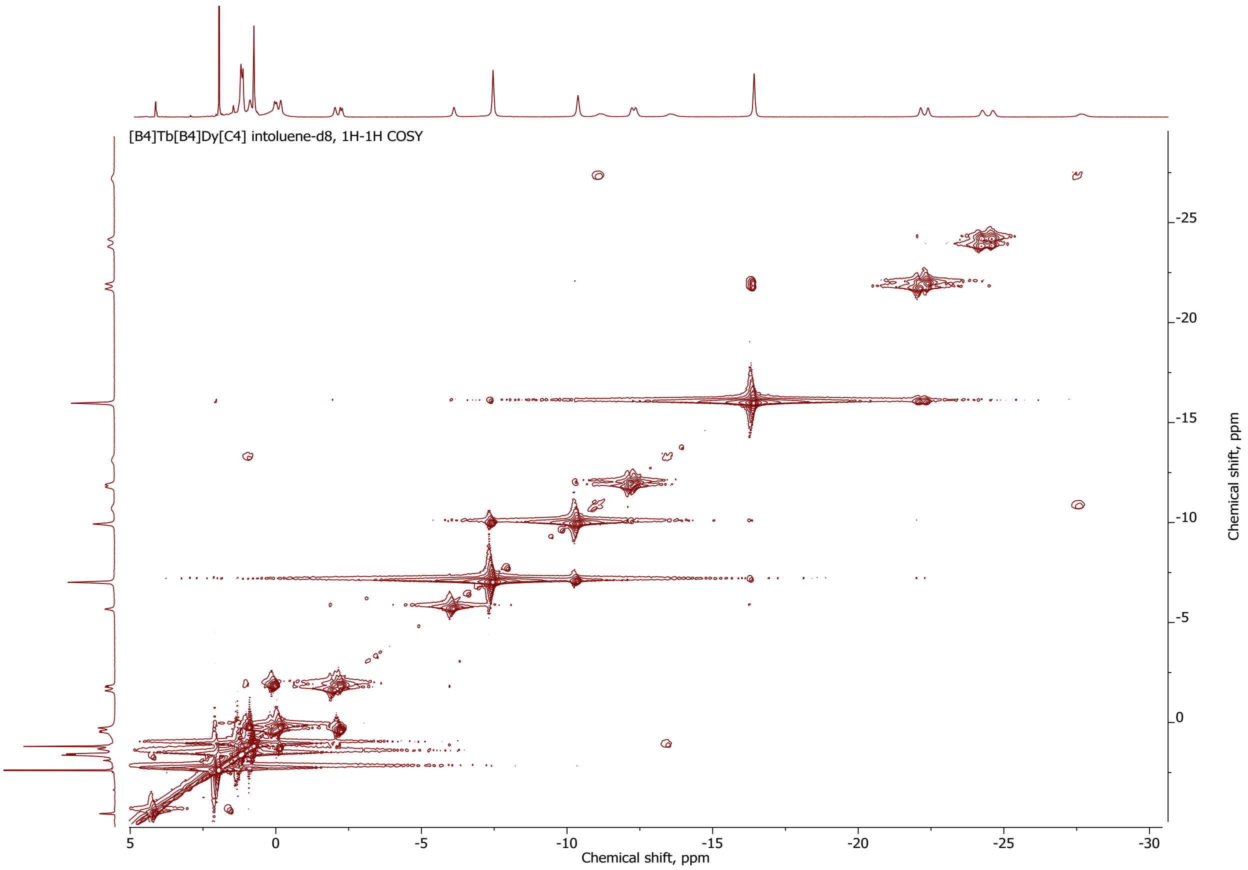

Figure S22. ^{1}H-^{1}H COSY spectrum of **[B$_4$]Tb[B$_4$]Dy[C$_4$]** in toluene-d$_8$.

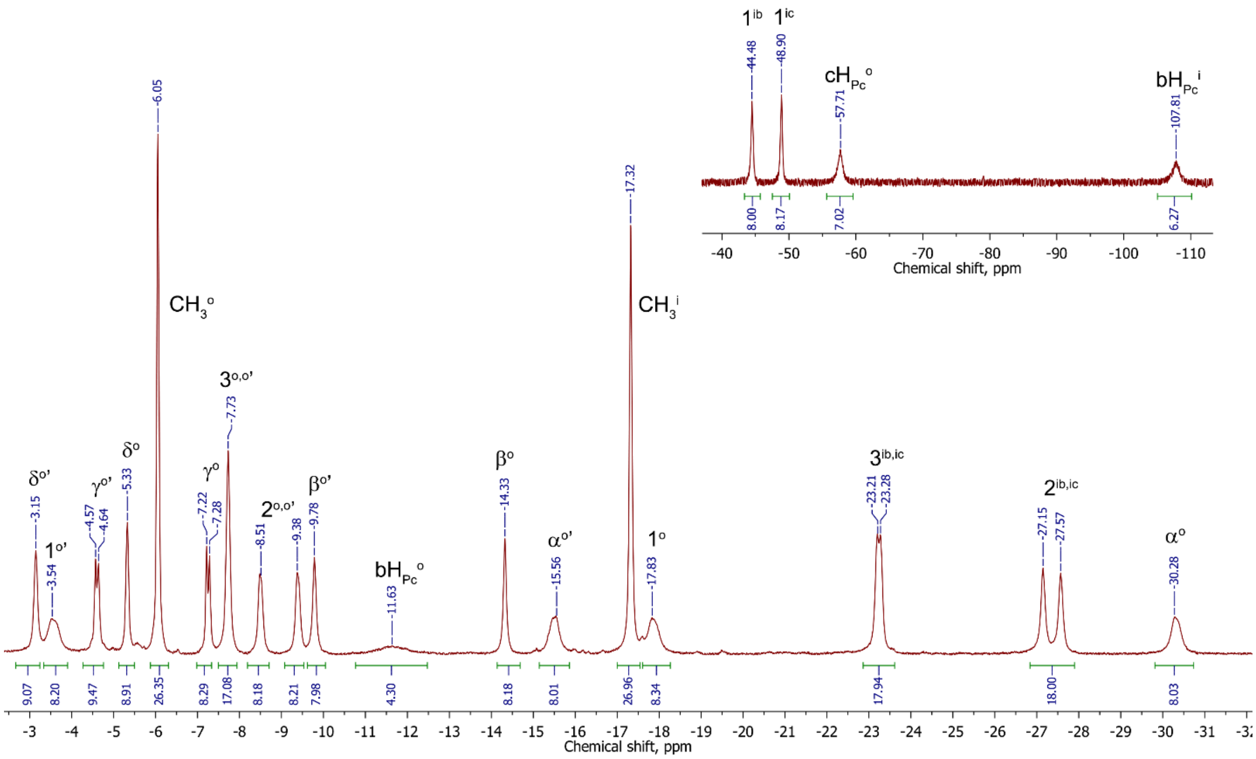

Figure S23. ^{1}H-NMR spectrum of **[B$_4$]Dy[B$_4$]Tb[C$_4$]** in $CDCl_3$.

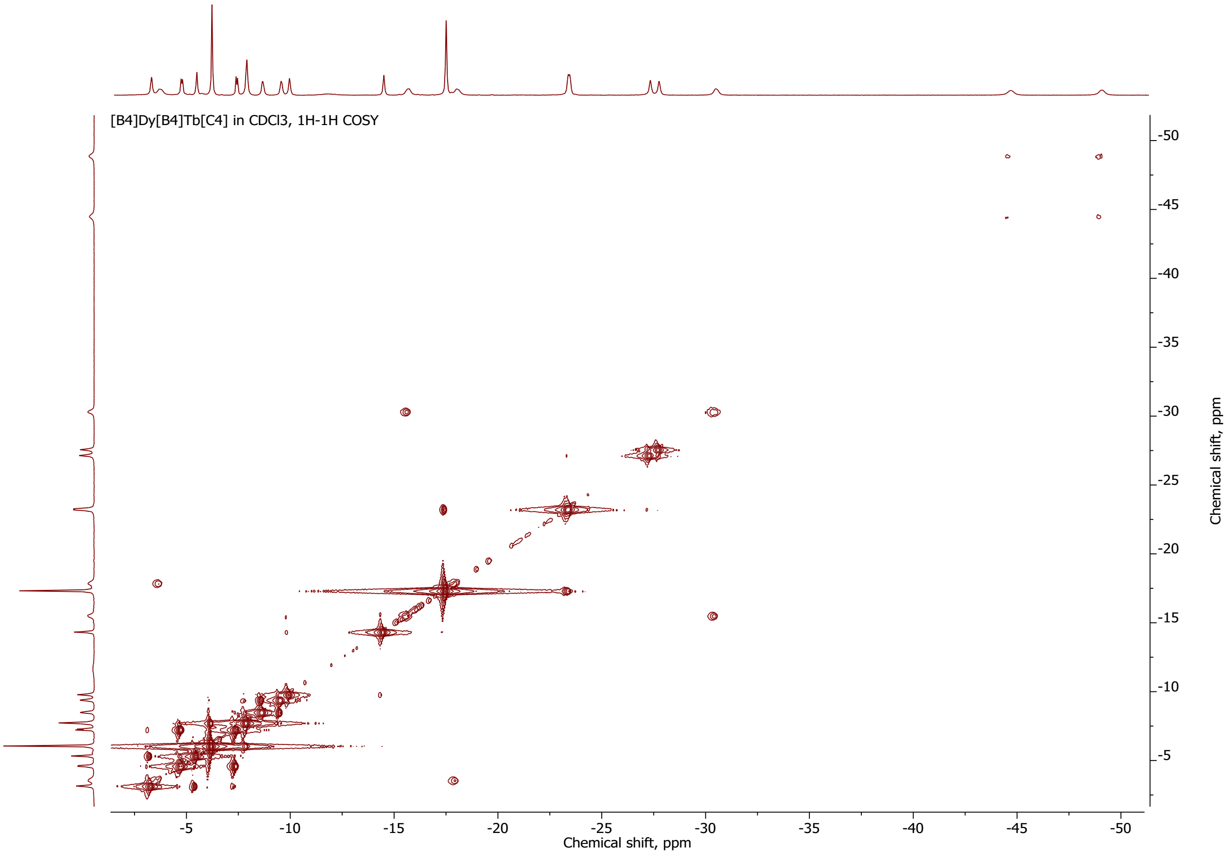

Figure S24. ^{1}H-^{1}H COSY spectrum of **[B$_4$]Dy[B$_4$]Tb[C$_4$]** in $CDCl_3$.

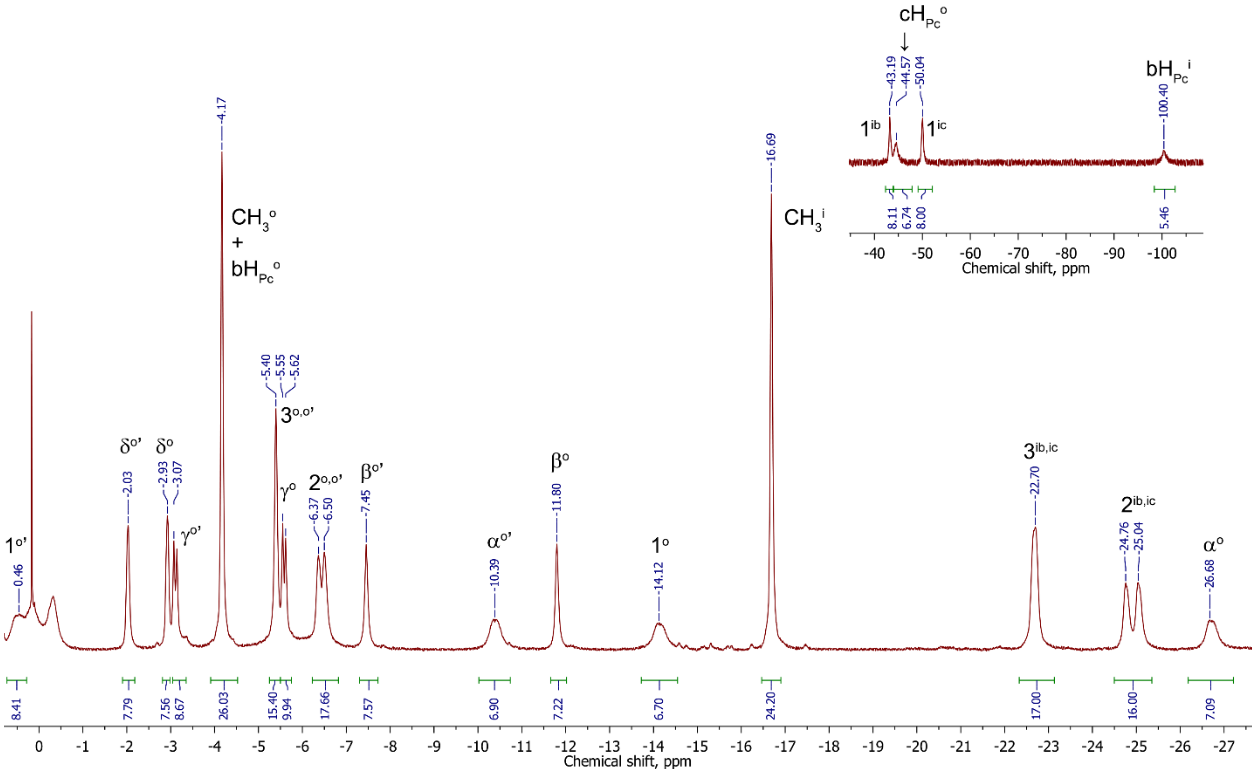

Figure S25. ^{1}H-NMR spectrum of **[B$_4$]Dy[B$_4$]Tb[C$_4$]** in toluene-d_8.

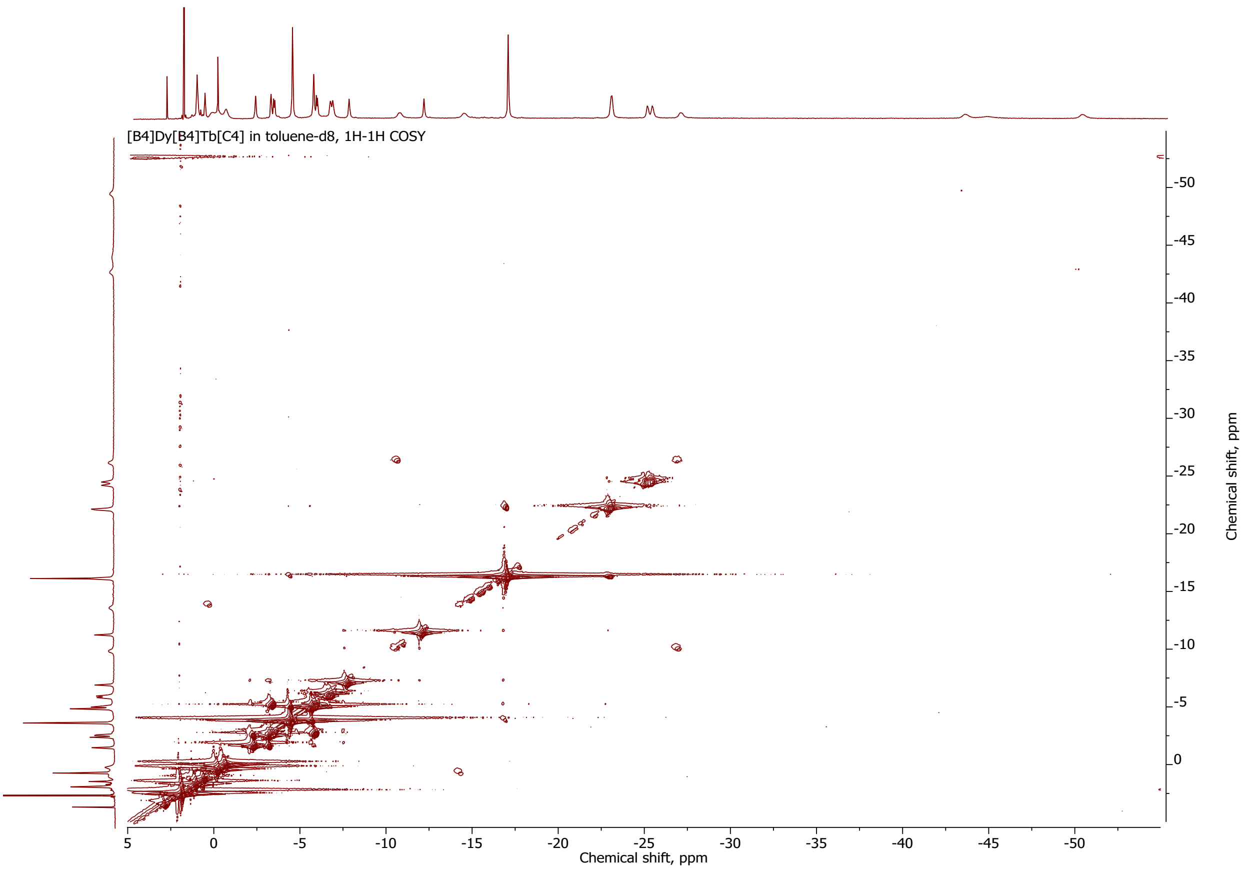

Figure S26. ^{1}H-^{1}H COSY spectrum of **[B$_4$]Dy[B$_4$]Tb[C$_4$]** in toluene-d_8.
